# Intracellular CYTL1, a novel tumor suppressor, stabilizes NDUFV1 to inhibit metabolic reprogramming in breast cancer

**DOI:** 10.1038/s41392-021-00856-1

**Published:** 2022-02-04

**Authors:** Wenwen Xue, Xin Li, Wuhao Li, Yixuan Wang, Chengfei Jiang, Lin Zhou, Jian Gao, Ying Yu, Yan Shen, Qiang Xu

**Affiliations:** 1 State Key Laboratory of Pharmaceutical Biotechnology, School of Life Sciences, Nanjing University, 210093 Nanjing, China; 2 Department of Pathology, Nanjing Medical University, 140 Hanzhong Road, 210029 Nanjing, China

**Keywords:** Cancer metabolism, Cell biology

## Abstract

Loss-of-function mutations frequently occur in tumor suppressor genes, i.e., p53, during the malignant progression of various cancers. Whether any intrinsic suppressor carries a rare mutation is largely unknown. Here, we demonstrate that intracellular cytokine-like protein 1 (CYTL1) plays a key role in preventing the robust glycolytic switching characteristic of breast cancer. A low intracellular CYTL1 level, not its mutation, is required for metabolic reprogramming. Breast cancer cells expressing an intracellular form of CYTL1 lacking a 1-22 aa signal peptide, ΔCYTL1, show significantly attenuated glucose uptake and lactate production, which is linked to the inhibition of cell growth and metastasis in vitro and in vivo. Mechanistically, CYTL1 competitively binds the N-terminal sequence of NDUFV1 to block MDM2-mediated degradation by the proteasome, leading to the stability of the NDUFV1 protein. In addition to inducing increased NAD^+^ levels, NDUFV1 interacts with Src to attenuate LDHA phosphorylation at tyrosine 10 and reduce lactate production. Our results reveal, for the first time, that CYTL1 is a novel tumor suppressor. Its function in reversing metabolic reprogramming toward glycolysis may be very important for the development of novel antitumor strategies.

## Introduction

An outstanding feature of cancer cell metabolism is the ability to switch from mitochondrial oxidative phosphorylation (OXPHOS) to aerobic glycolysis, even when sufficient oxygen is present.^1^ Coordinated upregulation of glycolysis, known as the Warburg effect, results in the production of both energy and intermediate metabolites that are required for the activation of biosynthetic pathways and the rapid proliferation of cancer cells.^2,3^ Increasing evidence has shown that, in most cancer cells, the causes of the Warburg effect are not attributable to mitochondrial dysfunction.^4^ In contrast, many mechanisms, including the activation of oncogenes and loss-of-function mutation of tumor suppressors, actively facilitate glycolytic switching.^5^ During this dominant trend toward malignancy, it is largely unknown whether there exists an intrinsic suppressor carrying a rare mutation to prevent the metabolic switching from OXPHOS to aerobic glycolysis in cancer.

Cytokine-like protein 1 (CYTL1) was originally identified in human CD34^+^ cells derived from bone marrow and cord blood. The definitive tertiary structure of the CYTL1 protein remains to be determined. Bioinformatics analysis suggests that CYTL1 has a signal peptide at its N-terminus from amino acid (aa) residues 1 to aa 22 and is characteristic of a secretory protein resembling interleukin (IL)-8-like chemokine folding.^6–8^ To date, CYTL1 has been shown to exert diverse biological functions, such as chemotactic and proangiogenic activities.^6,8^ In human neuroblastoma, high levels of CYTL1 expression were detected in tumor tissues and cell lines while silencing *cytl1* inhibited cell proliferation, migration, and invasion in SH-SY5Y neuroblastoma cells.^9^ In contrast to this report of CYTL1 in certain cell lines, CYTL1 was identified as one of seven gene signatures associated with a good prognosis in gastric cancer.^10^ A recent study reported that downregulation of *cytl1* expression was found in most types of tumors, including breast cancer, due to hypermethylation of *cytl1.*^11^ However, it remains unknown how CYTL1 acts in these diverse activities. These observations drove us to determine the function of CYTL1 in tumor progression.

In the present study, we reveal that CYTL1 may be a novel tumor suppressor that is capable of blocking the metabolic switching from OXPHOS to glycolysis. During metabolic reprogramming, tumor cells reduce the intracellular level of CYTL1, which has been shown to have a negative correlation with malignant progression in patients with breast cancer. Intracellular CYTL1 plays a previously unappreciated role in regulating metabolic reprogramming, dependent on NDUFV1, in breast cancer. NDUFV1 is a hydrophilic polypeptide found in the matrix arm of mitochondrial complex I (NADH:ubiquinone oxidoreductase) and binds the flavin cofactor that oxidizes NADH to NAD^+^.^12^ In contrast to known tumor suppressors involved in regulating cancer cell metabolism, which are frequently mutated, intracellular CYTL1 acts as a novel tumor suppressor that shifts metabolic reprogramming toward OXPHOS by stabilizing a mitochondrial protein in breast cancer.

## Results

### Low intracellular CYTL1 levels are required for metabolic reprogramming toward glycolysis in breast cancer cells

**Fig. 1:**
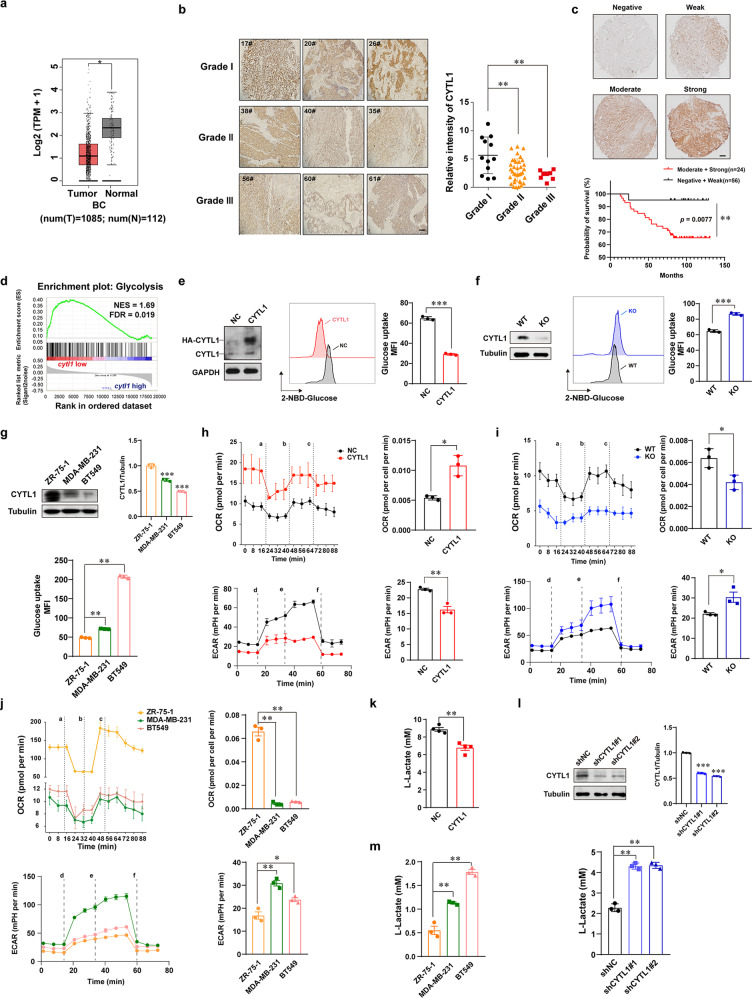
Loss of CYTL1 switches metabolism reprogramming to glycolysis in breast cancer. **a** Analysis of *cytl1* expression in human breast cancer issues (*n* = 1085) and adjacent normal tissues (*n* = 112) based on TCGA dataset. **b** The immunohistochemical images of breast cancer samples in different grades. Each grade shows three samples. The statistical relative intensity was evaluated by immunoreactivity intensities multiplied by the proportion of stain-positive cells, which was divided into 5 grades 0 (<5%), 1 (5–25%), 2 (26–50%), 3 (51–75%), and 4 (>75%). **c** Analysis of the relevance of CYTL1 protein expression with the survival rate of breast cancer patients. **d** Gene set enrichment analysis (GSEA) showing glycolysis gene signatures enriched in breast cancer patients with low *cytl1* expression relative to those with high *cytl1* expression based on TCGA database. Breast cancer samples were divided into *cytl1*-low and *cytl1*-high expression groups according to the median value. NES normalized enrichment score, FDR false discovery rate. **e** Glucose-uptake activity in MDA-MB-231 cells transfected with HA-tagged CYTL1 expressing plasmid was detected by flow cytometry. The protein levels of CYTL1 in the transfected cells were determined using anti-CYTL1 antibody by western blot (left panel). GAPDH was used as a loading control. **f** Glucose-uptake activity in CYTL1 KO cells. **g** Glucose-uptake ability in the indicated breast cancer cell lines with different levels of intact CYTL1 expression. The protein levels of CYTL1 in these cells were determined by western blot (upper panel). Tubulin was used as a loading control. The densitometry of the immunoblots was performed with the Image J software and is presented in the histograms. **h** The OCR (upper panel) and ECAR (lower panel) in MDA-MB-231 cells overexpressing CYTL1 were analyzed by the Seahorse. a, e: oligomycin, b: FCCP, c: antimycin, d: glucose, f: 2-DG. **i** The OCR (upper panel) and ECAR (lower panel) in CYTL1 KO cells. **j** The OCR (upper panel) and ECAR (lower panel) in the indicated breast cancer cell lines. **k**, **l** Lactate production levels in MDA-MB-231 cells transfected with **k** HA-tagged CYTL1 expressing plasmids or **l** the indicated shRNAs were detected by a spectrophotometer and normalized to the cell number. **m** Lactate production levels in the indicated breast cancer cell lines. The data are shown as the mean ± SD of three independent experiments. *P < 0.05, **P < 0.01, ***P < 0.001

To examine whether there is any novel tumor suppressor that is different from those currently known, we first performed bioinformatics analysis. The results showed that the level of *cytl1* expression was significantly lower in tumor tissues than in adjacent nontumor tissues in breast cancer patients based on data obtained from The Cancer Genome Atlas (TCGA) database (Fig. 1a). Compared with that in normal tissues, *cytl1* expression was lower in four different molecular subtypes of breast cancer (Supplementary Fig. S1a). Additionally, the TCGA database analysis revealed a decreasing trend in *cytl1* expression during breast cancer progression (Supplementary Fig. S1b). An immunohistochemical (IHC) analysis confirmed the negative correlation between CYTL1 protein expression and successive grades of breast cancer malignancy (Fig. 1b). The Gene Expression Omnibus (GEO) database analysis also showed that lower *cytl1* expression correlated with poor distant metastasis-free survival in breast cancer (Supplementary Fig. S1c). The level of *cytl1* expression was significantly lower in tumor tissues from breast cancer patients with metastasis than in those from the patients without metastasis (Supplementary Fig. S1d). Moreover, the analysis based on the GEO database showed that lower *cytl1* expression correlated with poor overall survival in breast cancer patients (Supplementary Fig. S1e). We then evaluated the relationship between the CYTL1 protein levels and the survival rate in 80 breast cancer patients using IHC, 56 patients with lower CYTL1 expression showed a significantly poor overall survival as compared with 24 patients with higher CYTL1 level (Fig. 1c). These findings suggest that lower CYTL1 protein expression is correlated with poor prognosis in breast cancer. Accordingly, the different levels of CYTL1 protein expression were confirmed in various breast cancer cell lines and most of them showed relatively low CYTL1 expression except ZR-75-1 cells (Supplementary Fig. S1f).

Because DNA hypermethylation may account for the low expression of CYTL1 in breast cancer,^11^ we evaluated the effect of the DNA-demethylating agent decitabine on CYTL1 expression in the MDA-MB-231 breast cancer cell line.^13^ Decitabine increased the mRNA expression of CYTL1 in a time-dependent manner (Supplementary Fig. S2a). Serving as a positive control, the gene expression of BRCA1, whose promoter methylation is associated with breast cancer induction,^14,15^ was measured and was found to be increased after decitabine treatment. In contrast to the robust increase in BRCA1 protein levels, the amount of CYTL1 protein in cell lysates was reduced after 4 days of decitabine treatment (Supplementary Fig. S2b). By performing an ELISA, we found that the amount of CYTL1 in the culture supernatant gradually increased after decitabine treatment (Supplementary Fig. S2c). These results suggest that breast cancer cells might avidly reduce the level of intracellular CYTL1 via an exocellular release.

In keeping with the previous studies,^11,16^ the TCGA database revealed a negative correlation between *cytl1* expression and its methylation levels in tumor tissues from breast cancer patients (Supplementary Fig. S3a, b). Interestingly, when the methylation levels of CYTL1 in breast cancer cell lines were analyzed based on the Cancer Cell Line Encyclopedia (CCLE), no relation was detected (Supplementary Fig. S3c). For example, the protein expression of CYTL1 in the ZR-75-1 cell line was the highest among 9 breast cell lines (Supplementary Fig. S1f), but its methylation level of CYTL1 was still high (Supplementary Fig. S3d). Because DNA copy number also has an important impact on the expression levels of the affected gene, we further analyzed CYTL1 copy number variance among breast cancer cell lines based on CCLE. Most breast cancer cell lines harbor low CYTL1 copy numbers (Supplementary Fig. S3e). The CYTL1 copy number in ZR-75-1 cells was the highest among 9 breast cell lines (Supplementary Fig. S3f). These findings support that breast cancer cells actively reduce both the mRNA and protein levels of CYTL1 to generate an intracellular environment with low CYTL1 expression in several methods.

To explore the mechanism by which CYTL1 regulates breast cancer progression, we performed transcriptome profiling using RNA-sequencing data obtained from the TCGA database. A gene set enrichment analysis revealed that the glycolytic gene signature was enriched in breast cancer patients with low *cytl1* expression (Fig. 1d, false discovery rate *q* = 0.019). Given that tumors reprogram metabolic pathways to meet the bioenergetic and biosynthetic demands of malignant cells,^4^ we investigated the effects of CYTL1 on aerobic glycolysis, which is characterized by an increase in glucose uptake and lactate production. Overexpressed CYTL1 led to reduced 2-NBD-glucose uptake by MDA-MB-231 cells, whereas CYTL1 deletion led to a significant increase in glucose uptake by CYTL1-knockout (KO) cells (Fig. 1e, f). Overexpressed CYTL1 reduced 2-NBD-glucose uptake in a dose-dependent manner (Supplementary Fig. S4a, b). We also compared glucose uptake by three representative breast cancer cell lines (Fig. 1g) and other six cell lines (Supplementary Fig. S4c) with different levels of intact CYTL1 expression. Consistently, glucose uptake activity was negatively associated with the expression level of CYTL1. ZR-75-1 cells with high CYTL1 levels exhibited a lower potential for glucose uptake than either MDA-MB-231 or BT549 cells with low CYTL1 levels (Fig. 1g). Moreover, CYTL1 overexpression in MDA-MB-231 cells augmented the basal oxygen consumption rate (OCR), whereas CYTL1 deletion attenuated the OCR increase (Fig. 1h, i). The results of the extracellular acidification rate (ECAR) were the opposite. Similar results were obtained with these three breast cancer cell lines (Fig. 1j), suggesting that CYTL1 may maintain OXPHOS status in breast cancer cells. Lactate is the final product of aerobic glycolysis.^2^ We found that CYTL1 overexpression reduced the abundance of extracellular lactate, whereas shRNA-mediated CYTL1 knockdown led to increased lactate production in MDA-MB-231 cells (Fig. 1k, l). The baseline levels of lactate production were also negatively associated with the levels of CYTL1 expression in the three breast cancer cell lines tested (Fig. 1m). Considering that CYTL1 is rarely mutated,^17^ our findings suggest that low intracellular levels of CYTL1, not loss-of-function mutation, are required for facilitating metabolic switching toward aerobic glycolysis.

### The intracellular form of CYTL1 inhibits metabolic reprogramming toward glycolysis

**Fig. 2:**
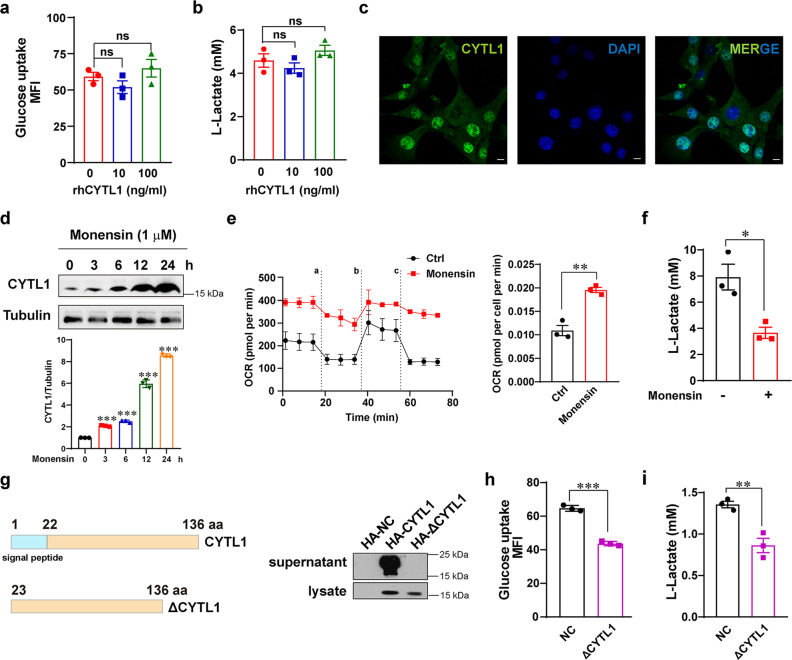
Intracellular CYTL1 inhibits glycolysis in breast cancer. **a** Glucose-uptake activity and **b** lactate production levels in MDA-MB-231 cells after treatment with rhCYTL1 at the indicated concentrations for 48 h. ns no significant. **c** The subcellular location of CYTL1 was analyzed by immunofluorescence in MDA-MB-231 cells. Representative data are shown from three independent experiments. Scale bar, 5 μm. **d** The amount of CYTL1 was determined by western blot in MDA-MB-231 cells after treatment with monensin for the indicated period. Tubulin was used as a loading control. **e** The OCR and **f** lactate production levels in MDA-MB-231 cells after treatment with 1 μM monensin for 24 h. **g** The supernatants and cell lysates from MDA-MB-231 cells transfected with HA-tagged CYTL1 or ΔCYTL1 expressing plasmids were determined by western blot using an anti-HA antibody. Right panel: schematic representation of the full-length CYTL1 and ΔCYTL1 plasmids. **h** Glucose-uptake activity and **i** lactate production levels in MDA-MB-231 cells overexpressing ΔCYTL1. The data are shown as the mean ± SD of three independent experiments. *P < 0.05, **P < 0.01, ***P < 0.001

Although numerous studies have focused on the function of CYTL1 as a secreted protein,^8^ low levels of CYTL1 mRNA and protein are associated with breast cancer progression and metabolic reprogramming to glycolysis, inspiring us to investigate the intracellular role of CYTL1. We abrogated the role of recombinant human CYTL1 (rhCYTL1) in glycolysis. Different concentrations of rhCYTL1 affected neither glucose absorption nor lactate production in MDA-MB-231 cells (Fig. 2a, b), supporting that the intracellular form of CYTL1, not the secreted form, regulates breast cancer cell metabolism. Based on human gene information available from the GeneCards database (https://www.genecards.org), a subcellular location of CYTL1 was predicted, in addition to its extracellular localization. Indeed, immunofluorescence assays showed that CYTL1 was distributed in both the cytoplasm and nucleus of MDA-MB-231 cells (Fig. 2c). When the secretion of CYTL1 was blocked by monensin, an inhibitor of trans-Golgi function, the amount of CYTL1 in the cell lysate was increased in a time-dependent manner, within 24 h (Fig. 2d). Concomitantly, the OCR was enhanced, but lactate production was profoundly reduced after 24 h of monensin treatment (Fig. 2e, f). When the expression of a C-terminal hemagglutinin (HA)-tagged full-length CYTL1 was analyzed in transfected MDA-MB-231 cells, the major form in supernatants displayed a molecular mass of about 20 kDa, which is distinguished from the 17 kDa form present only in cell lysates (Fig. 2g). This finding is similar to the data of Scheller et al.,^18^ indicative of the existence of two CYTL1 forms. To further address the possible role of the intracellular but not extracellular form of CYTL1 in breast cancer metabolism, we constructed a plasmid encoding ΔCYTL1, in which the 1-22 aa signal peptide of CYTL1 is lacking (Supplementary Fig. S5).^8,18^ In contrast to full-length CYTL1, ΔCYTL1 displayed size of about 17 kDa, but was not detected in the supernatant, suggesting complete inhibition of CYTL1 secretion. Ectopic ΔCYTL1 expression impaired MDA-MB-231 cell glucose absorption (Fig. 2h). The abundance of extracellular lactate was also reduced upon ΔCYTL1 expression (Fig. 2i). CCR2 (C-C chemokine receptor type 2) has been identified as a likely receptor for CYTL1, which mediates the extracellular signal-regulated kinase (ERK) signaling pathway.^19^ However, we failed to observe the effect of ΔCYTL1 on the mRNA expression of CCR2B or the activation of the following ERK signaling pathway (Supplementary Fig. S6), supporting that the intracellular CYTL1 may have a different function from the secreted form.

### The intracellular form of CYTL1 potently inhibits tumor growth and metastasis by impairing glycolysis

**Fig. 3:**
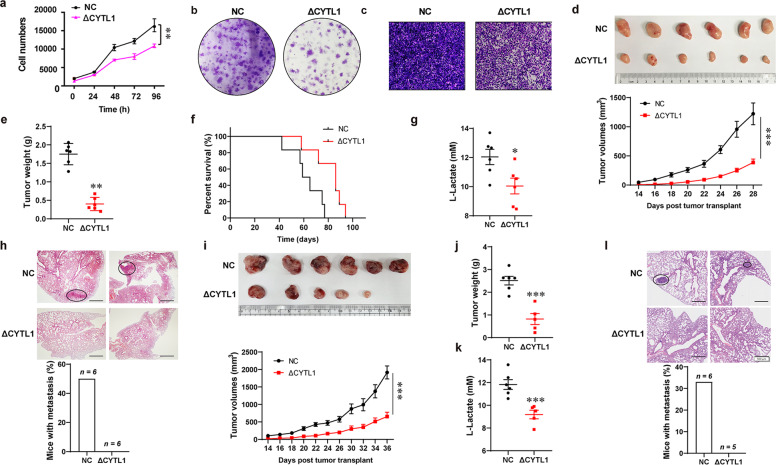
Intracellular CYTL1 prevents tumor growth and metastases. **a** Cell numbers were counted using a Trypan exclusion assay after MDA-MB-231 cells expressing ΔCYTL1 were cultured for the indicated period. **b** Colony formation assay was performed after MDA-MB-231 cells expressing ΔCYTL1 were cultured for 15 days. **c** Cell migration of MDA-MB-231 cells expressing ΔCYTL1 was determined using transwell assay. **d**–**h** Female nude mice were subjected to orthotopic injection with MDA-MB-231 cells stably expressing ΔCYTL1. Some mice were sacrificed 28 days later, after which tumors, lungs, and sera were collected, followed by photography and measurement. Some mice were monitored until they died. **d** Tumor growth curves (*n* = 6). Upper panel: representative images of the tumors at the end of the experiments. **e** Tumor weight. **f** Survival curve of mice (*n* = 6). **g** Serum lactate levels. **h** Representative H&E-stained lung sections. Circles indicate metastatic foci in the lungs. Scale bar, 500 μm. Lower panel: the proportion of mice with lung metastases in each group. **i**–**l** Female C57BL/6 mice were subjected to orthotopic injection with E0771 cells stably expressing ΔCYTL1. The mice were sacrificed 36 days later, after which tumors, lungs, and sera were collected. **i** Tumor growth curves (*n* = 6). Upper panel: representative images of the tumors at the end of the experiments. **j** Tumor weight. **k** Serum lactate levels. **l** Representative H&E-stained lung sections. Circles indicate metastatic foci in the lungs. Scale bar, 500 μm. Lower panel: the proportion of mice with lung metastases in each group. The data are shown as the mean ± SD of three independent experiments. *P < 0.05, **P < 0.01, ***P < 0.001

Next, we investigated whether the intracellular form of CYTL1 is capable of slowing or inhibiting the growth or metastasis of breast cancer cells in vitro or in vivo by targeting glycolysis. Ectopic ΔCYTL1 expression significantly inhibited MDA-MB-231 cell proliferation, colony formation, and invasion (Fig. 3a–c), suggesting a positive correlation between aerobic glycolysis and cancer cell growth.^4^ Then, MDA-MB-231 cells stably expressing ΔCYTL1 were inoculated into the mammary fat pad of female nude mice to establish an orthotopic transplant model (Supplementary Fig. S7a, b). Compared with the control, tumors formed by ΔCYTL1-transfected cells grew remarkedly slowly (Fig. 3d). The tumor weight was reduced by approximately threefold 28 days after implantation (Fig. 3e), and the overall mouse survival was prolonged (Fig. 3f). To evaluate the effect of expressing the intracellular form of CYTL1 on aerobic glycolysis in vivo, we measured serum lactate levels, which indicate the glycolytic status of mice.^20,21^ The mice bearing tumors expressing ΔCYTL1 showed lower levels of serum lactate than the control group, suggesting a lower glycolytic rate (Fig. 3g). Hematoxylin–eosin (H&E) staining showed no pulmonary metastasis 28 days after inoculation of MDA-MB-231 cells expressing ΔCYTL1, whereas the control group showed an increased incidence of metastasis of 50% (Fig. 3h). Similarly, tumor suppression was also observed in immunocompetent mice inoculated with E0771 murine breast adenocarcinoma cells stably expressing ΔCYTL1 (Fig. 3i, j and Supplementary Fig. S7c, d). Consistent with the findings above, much lower levels of serum lactate were detected in mice bearing tumor cells expressing ΔCYTL1 (Fig. 3k). In addition, H&E staining showed no pulmonary metastasis 36 days after inoculation of E0771 cells expressing ΔCYTL1, whereas the control group showed an increased incidence of metastasis of greater than 33% (Fig. 3l). However, no significant increase of the percentage of macrophages in the tumors formed by ΔCYTL1-transfected cells compared with control tumors (Supplementary Fig. S8), implying that ΔCYTL1 has no chemotactic effect on macrophages in mice. When the expression of the angiogenic gene in tumor tissues was examined, immunofluorescence revealed that CD31^+^ blood vessels in tumors formed by ΔCYTL1-transfected cells were significantly reduced as compared with control tumors (Supplementary Fig. S9), suggesting that ΔCYTL1 inhibited angiogenesis that is closely related to metastasis. Since glycolysis is involved in positive feedback to regulate STAT3 activity,^22^ we examined the effect of CYTL1 on the phosphorylation of STAT3. In keeping with the previous study,^11^ ectopic expression levels of both CYTL1 and ΔCYTL1 decreased the levels of p-STAT3 at Y705 (Supplementary Fig. S10). These results demonstrate that intracellular CYTL1 may function as a tumor suppressor via inhibiting glycolysis.

### Intracellular CYTL1 positively increases NDUFV1 protein expression and facilitates OXPHOS metabolism

**Fig. 4:**
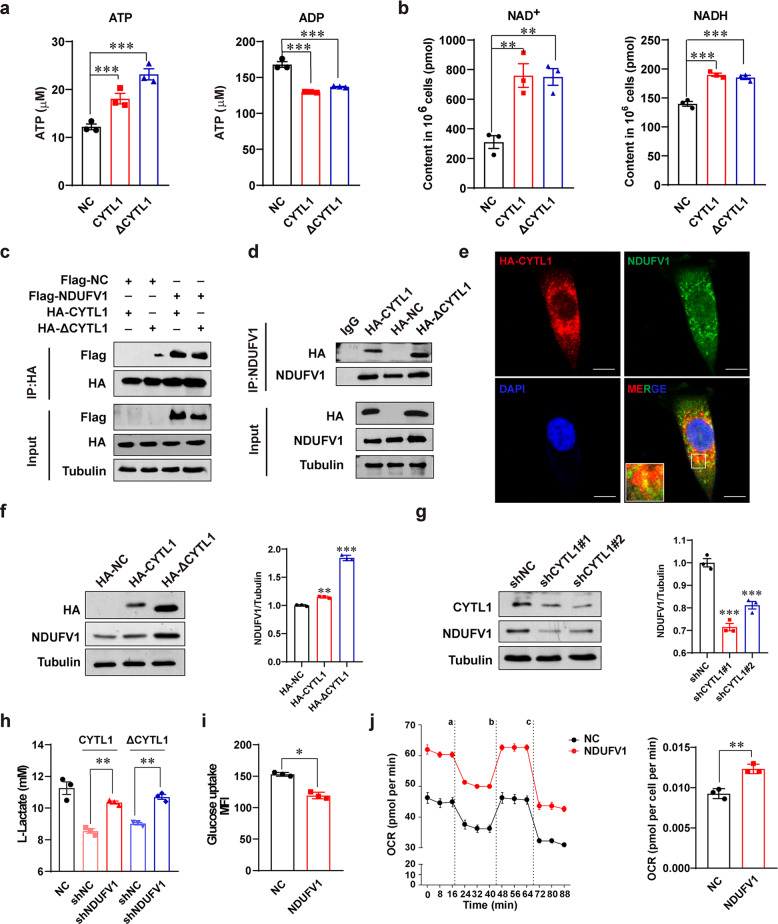
Intracellular CYTL1 interacts with NDUFV1 and positively regulates its protein expression. **a** ATP and ADP levels and **b** NAD^+^ and NADH levels in the cell lysates from MDA-MB-231 cells stably expressing CYTL1 or ΔCYTL1 were determined using kit assays. **c** Co-IP analysis of the interaction between exogenous HA-CYTL1 or HA-ΔCYTL1 and exogenous Flag-NDUFV1 in transfected HEK293T cells. **d** Co-IP analysis of the interaction between endogenous NDUFV1 and exogenous HA-CYTL1 or HA-ΔCYTL1 in transfected HEK293T cells. IgG served as the negative control. Tubulin was used as a loading control. **e** Colocalization of HA-CYTL1 and endogenous NDUFV1 was detected by Immunofluorescence in transfected HEK293T cells. Scale bar, 5 μm. **f**, **g** NDUFV1 protein expression was determined by western blot in MDA-MB-231 cells transfected with **f** HA-CYTL1 or HA-ΔCYTL1 expressing plasmids or **g** the indicated shRNAs. Tubulin was used as a loading control. **h** Lactate production levels in CYTL1 or ΔCYTL1 overexpressing MDA-MB-231 cells cotransfected with the indicated shRNAs. **i** Glucose-uptake activity and **j** the OCR in MDA-MB-231 cells overexpressing NDUFV1. The data are shown as the mean ± SD of three independent experiments. *P < 0.05, **P < 0.01, ***P < 0.001

To elucidate the mechanism by which intracellular CYTL1 regulates metabolic switching between OXPHOS and aerobic glycolysis in breast cancer cells, we examined the effect of CYTL1 on the production of pyruvate, which is the critical metabolite of the glycolytic pathway and crucial for the formation of lactate. No change in pyruvate content was observed in MDA-MB-231 cells stably expressing either CYTL1 or ΔCYTL1, as compared with that in control cells (Supplementary Fig. S11a). In contrast, ectopic expression of both forms of CYTL1 increased ATP and NAD^+^ production (Fig. 4a, b), suggesting the possibility that intracellular CYTL1 enhanced the oxidation of NADH to NAD^+^, depending on complex I in the mitochondrial electron transport chain. Using IACS-010759 as a positive control,^23^ we failed to detect a direct effect of rhCYTL1 on complex I activity (Supplementary Fig. S11b). We then performed pull-down experiments coupled with mass spectrometry to identify CYTL1-interacting proteins. NDUFV1 was a candidate among the 198 differentially interacting proteins (Supplementary Fig. S12). A coimmunoprecipitation (co-IP) analysis with HEK293T cells revealed that HA-tagged CYTL and ΔCYTL1 interacted with Flag-tagged NDUFV1 (Fig. 4c). Additionally, endogenous NDUFV1 interacted with both HA-CYTL1 and HA-ΔCYTL1 (Fig. 4d). Immunofluorescence confirmed the colocalization of HA-CYTL1 and NDUFV1 (Fig. 4e). Moreover, CYTL1 positively regulated NDUFV1 protein expression, as evidenced by the change in NDUFV1 protein levels in the case of CYTL1 overexpression or depletion (Fig. 4f, g and Supplementary Fig. S13). However, rhCYTL1 had no effect on NDUFV1 protein expression (Supplementary Fig. S14a), thus excluding a role of extracellular CYTL1. The mRNA level of NDUFV1 was not affected by CYTL1 abundance (Supplementary Fig. S14b, c), which excluded the possibility that CYTL1 affects the transcription of NDUFV1. Additionally, intact CYTL1 abundance positively correlated with NDUFV1 protein levels in the three breast cancer cell lines (Supplementary Fig. S14d). Moreover, silencing NDUFV1 reversed the reduction in lactate production caused by overexpressed CYTL1 or ΔCYTL1 in MDA-MB-231 cells, suggesting that CYTL1 inhibits glycolysis to maintain metabolic homeostasis in an NDUFV1-dependent manner (Fig. 4h). Consistently, NDUFV1 overexpression reduced glucose uptake and increased the OCR (Fig. 4i, j).

### CYTL1 stabilizes the NDUFV1 protein by reducing its mouse double minute 2 (MDM2)-mediated degradation

**Fig. 5:**
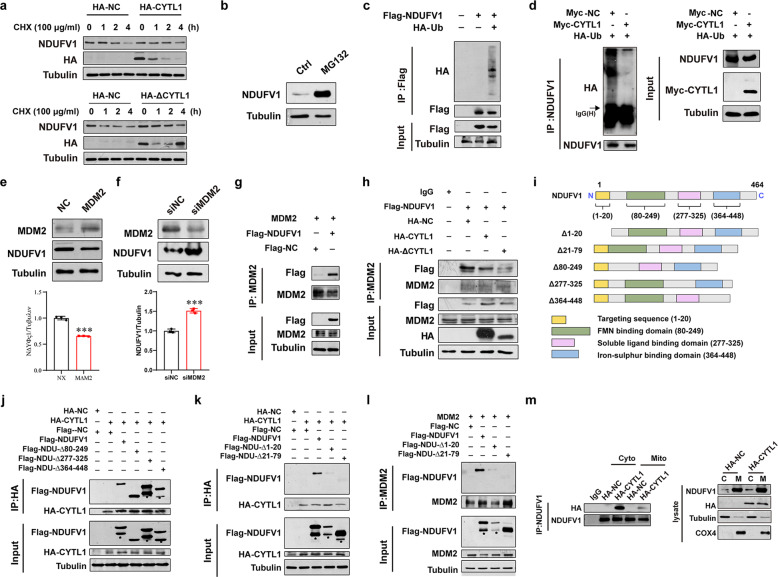
Intracellular CYTL1 interrupts MDM2-mediated degradation of NDUFV1 by competitive binding. **a** The degradation of NDUFV1 over time in the presence of cycloheximide (CHX) was monitored by western blot in MDA-MB-231 cells transfected with HA-CYTL1 or HA-ΔCYTL1 expressing plasmids. **b** The NDUFV1 protein level in MDA-MB-231 cells before and after treatment of 20 μM MG132 for 6 h. **c** Co-IP analysis of the interaction between Flag-NDUFV1 and HA-Ub in transfected HEK293T cells. **d** Co-IP analysis of the interaction between NDUFV1 and HA-Ub in HEK293T cells cotransfected with Myc-NC or Myc-CYTL1 after treatment of 20 μM MG132 for 6 h. **e**, **f** NDUFV1 protein expression was determined by western blot in MDA-MB-231 cells transfected with **e** MDM2 expressing plasmid or **f** siRNA specific for MDM2. Tubulin was used as a loading control. **g** Co-IP analysis of the interaction between MDM2 and Flag-NDUFV1 in transfected HEK293T cells. **h** Co-IP analysis of the interaction between MDM2 and Flag-NDUFV1 in transfected HEK293T cells cotransfected with HA-NC, HA-CYTL1, or HA-ΔCYTL1. **i** Schematic representation of NDUFV1 mutants that have truncated sequences. **j** Co-IP analysis of the interaction between HA-CYTL1 and Flag-NDUFV1 or its three mutants in transfected HEK293T cells. Stars indicate non-specific bands. **k** Co-IP analysis of the interaction between HA-CYTL1 and Flag-NDUFV1 or its two mutants with deletion of N-terminal sequences. **l** Co-IP analysis of the interaction between MDM2 and Flag-NDUFV1 or its two mutants. **m** Cytoplasmic and mitochondrial protein were extracted from HEK293T cells transfected with HA-NC or HA-CYTL1. Co-IP analysis was performed for the interaction between endogenous NDUFV1 and HA-CYTL1. The data are shown as the mean ± SD of three independent experiments. ***P < 0.001

Next, we monitored the degradation of the NDUFV1 protein by using the protein synthesis inhibitor cycloheximide (CHX) to treat MDA-MB-231 cells stably expressing HA-tagged CYTL1 or ΔCYTL1. The NDUFV1 protein was degraded rapidly in the control cells treated with CHX, within 4 h (Fig. 5a). However, the ectopic expression of CYTL1 or ΔCYTL1 delayed NDUFV1 degradation. Treatment of MDA-MB-231 cells with the proteasome inhibitor MG132 increased NDUFV1 protein levels (Fig. 5b). A Co-IP analysis verified the interaction between NDUFV1 and ubiquitin (Ub) (Fig. 5c). This interaction was blocked in cells with ectopically expressed CYTL1 (Fig. 5d). These results suggest that the NDUFV1 protein is protected in a CYTL1-dependent manner from degradation by the Ub–proteasome system.

MDM2 is an E3 Ub ligase that is overexpressed in multiple cancer types, including breast cancer.^24,25^ To test the possibility that MDM2 mediates the ubiquitination of NDUFV1, we examined the impact of MDM2 expression on NDUFV1 protein levels. MDM2 overexpression reduced the level of the NDUFV1 protein (Fig. 5e), whereas small interfering RNA (siRNA)-mediated MDM2 knockdown resulted in the opposite effect (Fig. 5f). When exogenous MDM2 was immunopurified from HEK293T cells and probed with Flag-tagged NDUFV1, the MDM2-NDUFV1 interaction was detected and was partially blocked by overexpressed CYTL1 or ΔCYTL1 (Fig. 5g, h), indicating that CYTL1 may interrupt MDM2-mediated degradation of NDUFV1 via competitive binding. We transfected HEK293T cells with the indicated truncated versions of NDUFV1 (Fig. 5i). Although the deletion of three predicted functional structural domains of NDUFV1 (the FMN-binding domain, soluble ligand-binding domain, and iron–sulfur-binding domain, http://www.ebi.ac.uk/interpro/protein/UniProt/P49821) had no effect on the NDUFV1 interaction with CYTL1, 1–20 aa (a mitochondrial localization sequence) and 21–79 aa domain of NDUFV1 were required for its binding to both CYTL1 and MDM2 (Fig. 5j–l). In the cytosol, there is normally minimal NDUFV1, as once this nuclear-encoded mitochondrial protein is transcribed and translated, NDUFV1 is imported into mitochondria. And MDM2-mediated proteasomal degradation frequently occurs within the cytosol.^26^ Indeed, we noticed a preferential interaction between NDUFV1 and CYTL1 in the cytosol (Fig. 5m). These results suggest that intracellular CYTL1 competitively binds NDUFV1 to prevent its degradation as mediated by MDM2 in the cytosol.

### NDUFV1 reduces lactate dehydrogenase A (LDHA) activity by suppressing LDHA Y10 phosphorylation

**Fig. 6:**
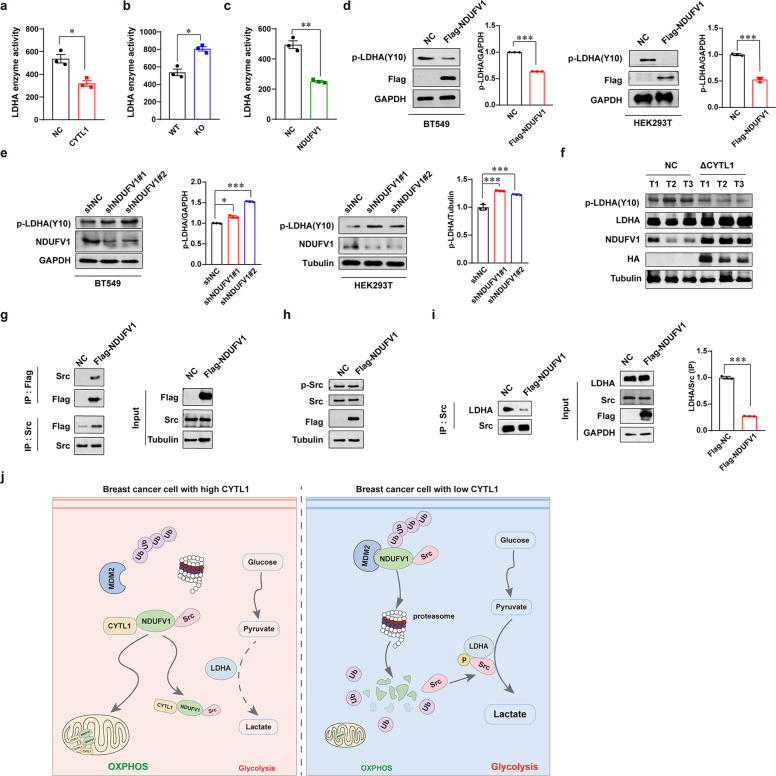
NDUFV1 suppresses the Y10 phosphorylation of LDHA by Src. **a** LDHA enzyme activity in MDA-MB-231 cells overexpressing CYTL1 was detected using kit assay. **b** LDHA enzyme activity in CYTL1 KO cells. **c** LDHA enzyme activity in MDA-MB-231 cells overexpressing NDUFV1. **d**, **e** LDHA Y10 phosphorylation level was determined by western blot in the indicated cells transfected with **d** Flag-NDUFV1 expressing plasmids or **e** the shRNAs against NDUFV1. GAPDH was used as a loading control. **f** MDA-MB-231 orthotopic breast cancer models were established as described in Fig. 4. The indicated proteins in tumor tissues were determined by western blot. **g** Co-IP analysis of the interaction between Flag-tagged NDUFV1 and endogenous Src in transfected HEK293T cells. **h** The levels of phosphorylated Src and total Src were determined by western blot in MDA-MB-231 cells overexpressing NDUFV1. **i** Co-IP analysis of the interaction between Src and LDHA in HEK293T cells transfected with Flag-NDUFV1 expressing plasmids. **j** A schematic model for the role of intracellular CYTL1 in regulating metabolic reprogramming in breast cancer. The data are shown as the mean ± SD of three independent experiments. *P < 0.05, **P < 0.01, ***P < 0.001

LDHA, a critical metabolic enzyme in glycolysis, catalyzes the interconversion of pyruvate and lactate and promotes tumor progression.^27^ Given the significant reduction in lactate production in breast cancer cells overexpressing CYTL1, we investigated whether intracellular CYTL1 influences LDHA enzymatic activity by stabilizing NDUFV1. As shown in Fig. 6a, b, CYTL1 abundance was negatively related to LDHA activity. Similarly, NDUFV1 overexpression also inhibited LDHA activity (Fig. 6c). However, LDHA protein levels were not affected by the abundance of CYTL1, ΔCYTL1, or NDUFV1 (Supplementary Fig. S15). Since LDHA activity is frequently attributed to its phosphorylation at tyrosine 10,^28,29^ we next examined the effect of NDUFV1 on LDHA phosphorylation. Overexpression of NDUFV1 inhibited LDHA Y10 phosphorylation in both BT549 and HEK293T cells, whereas knockdown of NDUFV1 resulted in the opposite effect (Fig. 6d, e). In fact, a profound reduction in LDHA Y10 phosphorylation levels was detected in the tumor tissues expressing ΔCYTL1 in the orthotopic breast cancer model described in Fig. 4 (Fig. 6f). We then performed pull-down experiments coupled with mass spectrometry to identify NDUFV1-interacting proteins. Src was a candidate among the 449 differentially interacting proteins (Supplementary Fig. S16). A previous study reported that Src phosphorylates LDHA at tyrosine 10 in breast cancer.^30^ And shRNA of CYTL1 or NDUFV1 led to a significant increase in lactate production in MDA-MB-231 cells, which was completely reversed by the Src kinase inhibitor KX2-391 (Supplementary Fig. S17). Our co-IP analysis further confirmed that Flag-tagged NDUFV1 interacted with endogenous Src (Fig. 6g). Notably, overexpressing NDUFV1 had no effect on Src phosphorylation, indicating that NDUFV1 was not able to stimulate Src activation (Fig. 6h). Another co-IP analysis showed that NDUFV1 interfered with the binding of Src to LDHA (Fig. 6i). These results suggest that NDUFV1 can interact with Src to inhibit LDHA phosphorylation. Taken together, intracellular CYTL1 may be a novel tumor suppressor (Fig. 6j). It actively facilitates metabolic switching toward OXPHOS by stabilizing NDUFV1 that is degraded via the MDM2-mediated proteasomal system and is capable of interacting with Src to inhibit LDHA activity. In breast cancer with low CYTL1 levels, cancer metabolic reprogramming is switched from OXPHOS to glycolysis.

## Discussion

As a cytokine-like protein, secreted CYTL1 has received considerable attention. In this study, we demonstrate, for the first time, that the intracellular form of CYTL1, not the extracellular form, inhibits metabolic reprogramming toward glycolysis, which has been linked to the inhibition of the cell growth and metastasis of breast cancer both in vitro and in vivo. These findings highlight CYTL1 as a novel tumor suppressor. Cancer is identified by various hallmarks that indicate its malignant progression, including robust facilitation of glycolytic switching.^5^ For this metabolic switching, oncogenes are typically activated and tumor suppressors are inactivated in tumors. In the case of breast cancer, a negative correlation between malignant progression and the intracellular level of CYTL1 was observed. The trend of profound reduction in CYTL1 level in advanced tumors suggests the possibility that CYTL1 can be recognized as a suppressor that hinders tumor progression. In contrast to suppressors known to be prone to mutation, i.e., p53 and PTEN, to our knowledge, CYTL1 harbors rare loss-of-function variants.^17,31^ These features of CYTL1 as a novel tumor suppressor may be very important not only for our understanding of tumor suppressors but also for the development of new antitumor approaches.

Through bioinformatics analyses and experimental confirmation by using clinical samples and cell lines, the reduction of CYTL1 level and its negative relationship with clinic prognosis were uncovered in breast cancer. CYTL1 can be reduced in several methods. Apart from DNA hypermethylation, active exocellular release and loss of DNA copy number may account for the low expression of CYTL1 during the malignant progression of breast cancer. It is possible that breast cancer cells might actively reduce both the mRNA and protein levels of CYTL1 to generate an intracellular environment with low CYTL1 expression.

Given that tumors reprogram metabolic pathways to meet the bioenergetic and biosynthetic demands of malignant cells,^4^ we investigated the effects of CYTL1 on aerobic glycolysis, including glucose uptake, OCR, ECAR, and lactate production. The results indicate that CYTL1 may maintain OXPHOS status and low intracellular levels of CYTL1 in breast cancer cells are indispensable for facilitating metabolic switching toward aerobic glycolysis. Moreover, the regulator for breast cancer cell metabolism was identified to be the intracellular form rather than the secreted form of CYTL1, because ΔCYTL1 lacks the 1-22 aa signal peptide, but not rhCYTL1, affected glycolysis. Notably, ectopic ΔCYTL1 expression significantly inhibited breast cancer cell proliferation and invasion, and STAT3 activation slowed the tumor growth and prolonged the survival without pulmonary metastasis in animal models. More importantly, lower levels of serum lactate were detected in the mice bearing tumors expressing ΔCYTL1. These results suggest that intracellular CYTL1 may function as a tumor suppressor via inhibiting glycolysis in breast cancer.

According to the increase in ATP and NAD^+^ induced by overexpressed CYTL1, we reasoned that CYTL1 may enhance the oxidation of NADH to NAD^+^, depending on complex I in the mitochondrial electron transport chain. Indeed, mass spectrometry analysis revealed the complex I subunit NDUFV1 as a candidate of CYTL1-interacting proteins. Co-IP analyses and immunofluorescence confirmed the interaction of CYTL1 and NDUFV1. And intracellular CYTL1 was found to protect NDUFV1 degradation via the Ub-proteasomal system. A recent study reported that cytosolic MDM2 could directly bind and sequester NDUFS1 (NADH-ubiquinone oxidoreductase 75 kDa subunit 1, an Fe-S protein), another component of complex I.^26^ Similar to NDUFS1, NDUFV1 bound the E3 Ub ligase MDM2 through a 1-79 aa sequence in the NDUFV1 N-terminus. This binding was competitively blocked by CYTL1, and NDUFV1 protein degradation was inhibited in the presence of CYTL1. Despite its main location in mitochondria, NDUFV1 preferentially interacted with CYTL1 in the cytosol. Thus, intracellular CYTL1 protects NDUFV1 from MDM2-mediated degradation via competitive binding to the N-terminal sequence of NDUFV1. The CYTL1 expression variance contributes to the variance in NDUFV1 expression in breast cancer cells. And the blockade of NDUFV1 degradation in the cytosol may facilitate its mitochondrial localization and maintain OXPHOS status.

Glycolysis is under the negative control of OXPHOS. Cell models with mitochondrial complex I deficiency display activation of glycolysis, including an increase in lactate production and glycolytic flux.^32,33^ Additionally, genetic or pharmacological OXPHOS inhibition results in compensatory upregulation of glycolysis to maintain ATP levels and redox balance.^34^ Consistently, we found that in addition to increasing NAD^+^ production, NDUFV1 remarkably inhibited Y10 LDHA phosphorylation to negatively control glycolysis. Moreover, CYTL1-mediated glycolysis inhibition was dependent on NDUFV1. Recent studies have shown that the plasma membrane hosts many mitochondrial proteins, including OXPHOS complex subunits.^35,36^ The noncanonical functions of NDUFV1 outside mitochondria remain to be further investigated.

Although increased glycolysis in cancer cells is very important, efforts to target glycolytic metabolism in clinical trials have met with little success.^2,37^ This failure reflects the metabolic plasticity of cancer cells and their tendency to escape glucose dependency through other metabolic pathways. In addition, increasing evidence has shown that mitochondrial metabolism is vital for tumor growth.^23,38^ Therefore, new therapeutic strategies are required to overcome the metabolic plasticity of cancer cells.

Accumulating evidence indicates that the intracellular forms of some secreted proteins, such as IL-33, IL-37, and MIF (macrophage migration inhibitory factor), are involved in the biological processes via interacting with cytoplasmic proteins.^39–41^ Also, CYTL1 has received considerable attention as a secreted cytokine-like protein. This study demonstrates that the intracellular form of CYTL1 has a distinctive function from the secreted form. The reason why these secreted proteins retain within cells is still unknown. The possibility includes alternative translation initiation, retrotranslocation from the endoplasmic reticulum into the cytoplasm and/or incomplete targeting to secretory vesicles. To further investigate the function of the intracellular form of CYTL1 and the underlying mechanisms would facilitate a therapeutical approach.

Taken together, our study reveals the function of CYTL1 as an intrinsic tumor suppressor to maintain metabolic homeostasis in tumors, which is helpful by providing potential points of therapeutic intervention against cancers. Thus, regulating intracellular CYTL1 expression and its stabilization of the NDUFV1 protein might be a strategic approach for breast cancer therapy.

## Materials and Methods

### Reagents and antibodies

Oligomycin, carbonyl cyanide 4-(trifluoromethoxy) phenylhydrazone, antimycin, and CHX were purchased from Sigma-Aldrich (St. Louis, MO). Decitabine, monensin, MG132, KX2-391, and IACS-010759 were from Selleck (Houston, TX). Recombinant human CYTL1 was purchased from OriGene Technologies (Rockville, MD). Anti-CYTL1 antibody was purchased from Abcam (Cambridge, UK). Anti-NDUFV1, anti-BRCA1, anti-Flag, anti-GAPDH, and anti-Src antibodies were from Proteintech Group (Chicago, IL). Anti-HA, anti-CD31, anti-MDM2, anti-LDHA, anti-p-LDHA (Y10), and anti-COX4 antibodies were from Cell Signaling Technology (Beverly, MA). Anti-Tubulin, anti-β-Actin, anti-myc, and p-Src (Santa Cruz) antibodies were from Santa Cruz Biotechnology (Santa Cruz, CA). Lipofectamine 3000 and Lipofectamine RNAi MAX were purchased from Thermo Fisher Scientific (Waltham, MA). MitoCheck Complex I activity assay kit (No.700930) was purchased from Cayman Chemical (Ann Arbor, Michigan). Human CYTL1 ELISA Kit was purchased from CUSABIO Life Sciences (College Park, MD).

### Cell culture

Human breast cancer cell lines, including estrogen receptor (ER) positive ZR-75-1, T47D, MCF7, BT474; ER negative; triple negative Sum1315, BT549, MDA-MB-231, MDA-MB-468, HCC1937, and human embryonic kidney epithelial cell HEK293T were purchased from the Cell Bank Type Culture Collection of the Chinese Academy of Sciences (Shanghai), who provided an authentication certificate. Murine breast adenocarcinoma cell E0771 was kindly provided by professor Siying Wang (Anhui Medical University). MDA-MB-231, ZR-75-1, and HEK293T cells were cultured in DMEM (Life Technologies, Grand Island, NY), and BT549 cells were cultured in RPMI1640 medium (Life Technologies) containing 10% FBS (GIBCO, Grand Island, NY), 100 mg/ml streptomycin and 100 U/ml penicillin at 37 °C in a humidified atmosphere with 5% CO_2_.

### Mice

Six-to-8-week-old female BALB/c nude mice and C57BL/6 mice were obtained from the Model Animal Research Center of Nanjing University (Nanjing, China). All animal studies were performed in compliance with the guidelines (Ministry of Science and Technology of China, 2006) and relevant ethical regulations of Nanjing University. All efforts were made to minimize the suffering of animals and the number of animals used.

### Plasmids, CRISPR/Cas9-mediated KO, and RNA interference

The plasmids expressing human CYTL1 (RC206778) and mouse CYTL1 (MR221433) were obtained from OriGene Technologies. Full length of human CYTL1 and NDUFV1 were subcloned into vector CMV-MCS-HA-SV40-Neomycin and pLenti-EF1a-mcherry-P2A-Puro-CMV-MCS-3Flag, respectively. Fragment cDNAs of ΔCYTL1 lacking a 1–22 aa, Flag-NDU-Δ1-20, Flag-NDU-Δ21-79, Flag-NDU-Δ80-249, Flag-NDU-Δ277-325, Flag-NDU-Δ364-448 were generated by standard PCR and cloned into the restriction sites. The full length of human MDM2 was subcloned into vector pcDNA3.1(+). shCYTL1#1 and shCYTL1#2 were designed and cloned into vector pGPU6/Neo. shNDUFV1#1and shNDUFV1#2 were cloned into vector pGV248. A retroviral vector expressing HA-ΔCYTL1 was generated by Obio Technology (Shanghai, China.)

For the generation of CRISPER/Cas9 KO cell lines, single guide RNAs targeting CYTL1 were designed using CRISPR Design (http://crispr.mit.edu/) and cloned into the pYSY-CMV-Cas9-U6-gRNA-EFla-puromycin. MDA-MB-231 cells were transfected with the appropriate plasmid and screened in the presence of 2.5 μg/ml. Single KO clones were verified by immunoblotting and sequencing of the PCR fragments.

For RNA interference, siRNA was obtained from Gene Script (Nanjing) and transfected using Lipofectamine RNAi MAX. MDM2 siRNA sequence was 5′-GCACCUCACAGAUUCCAGC-3′. Luciferase siRNA was used as described previously.^42^

### Immunohistochemistry

The tissue microarray of human breast cancer (BR811) was purchased from US Biomax, Inc (Rockville, MD). A tissue microarray containing 80 paired human breast cancer tissues (Cat No. BRC1602) was purchased from the shanghai Superbiotek Pharmaceutical Technology Co., Ltd. (Shanghai). Paraffin-embedded sections were stained with the antibody against CYTL1 and then determined using the Real Envision Detection kit (GeneTech, Shanghai) according to the manufacturer’s instructions. Data analysis was performed blindly.

### Western blot, co-IP, cell viability, and colony formation assay

The protocols have been reported previously.^43^ For western blot and co-IP, the densitometry of the immunoblots was performed with the Image J software and is presented in the histograms. The data are shown as the mean ± SD of three independent experiments.

### Glucose uptake and lactate production assay

The glucose absorption was analyzed with a FACSCalibur flow cytometer (Becton Dickinson, San Jose, CA) by using Glucose Uptake Cell-Based Assay Kit (No.600470, Cayman Chemical). Lactate production was determined using Glycolysis Cell-Based Assay Kit (No.600450, Cayman Chemical) according to the manufacture’s protocol.

### Measurement of OCR and ECAR

OCR and ECAR were determined by using a Seahorse XF96 Bioanalyzer (Seahorse Bioscience) following the manufacture’s protocol.

### Immunofluorescence

Cells on coverslips were fixed with 4% paraformaldehyde for 30 min at room temperature, permeabilized with phosphate-buffered saline (PBS) containing 0.1% Triton X-100 for 30 min, blocked with 3% bovine serum albumin (BSA) for 1 h, and then incubated with the primary antibodies at 4 °C overnight. After washing three times with PBS, the cells were incubated with Alexa Fluor-conjugated secondary antibodies for 2 h. The nucleus was stained with 4,6-diamidino-2-phenylindole (Beyotime, Shanghai) for 2 min. After staining, the cells were prepared for microscopic analysis. Fluorescent images were acquired using the FluoView TM FV1000 confocal microscope (Olympus, Tokyo, Japan) and analyzed by the Olympus Fluview Ver1.7b viewer (Olympus).

### Transwell invasion assay

Cells were suspended at a density of 200,000 cells per ml in 0.5% BSA serum-free medium, and seeded into the upper chamber (0.1 ml) of 8 μM pore-size Transwell inserts (Corning, Lowell, MA). After 24 h, the cells on the upper surface of the membrane were removed using cotton tips. The migrant cells attached to the lower surface were fixed in 10% formalin at room temperature for 30 min and stained for 20 min with a solution containing 1% crystal violet and 2% ethanol in 100 mM borate buffer, pH 9.0.

### In vivo experiments

Two murine cancer models were established. In one model, MDA-MB-231 cells (2 × 10^6^ cells in 20 μl PBS) stably expressing HA-ΔCYTL1 were injected into the fourth mammary fat pad in the lower abdomen of the female nude mice (*n* = 12 mice per group). After 28 days, the mice were sacrificed. Tumor and blood were collected, followed by photography and measurement. Some mice were monitored daily until all the mice died to make a survival test (*n* = 6 mice per group). For another, E0771 cells (2 × 10^5^ cells in 20 μl PBS) stably expressing myc-ΔCYTL1 were injected into female C57BL/6 mice (*n* = 6 mice per group) as above. After 36 days, the mice were sacrificed. Tumor, blood, and lung tissues were collected. Tumor volumes were determined by caliper measurement every 2 days and calculated using the following formula: *V* = 0.5236 × L1 × (L2)^2^, where L1 is the longer and L2 is the shorter tumor axis.

### Histologic analysis

Lung tissues from mice were obtained for H&E staining.

### ADP and ATP detection assay

ADP and ATP production was determined using ADP Assay Kit (Colorimetric/Fluorometric) (ab83359, Cambridge, MA) and ATP Detection Assay Kit—Luminescence (No.700410, Cayman Chemical), respectively, according to the manufacture’s protocol. In brief, ATP levels are measured by luciferase/luciferin-mediated assay. For the measurement of ADP, ADP is converted to ATP and pyruvate. Then the generated pyruvate is quantified by the colorimetric method.

### NAD^+^ and NADH detection assay

NAD^+^ and NADH production was determined using NAD^+^/NADH Detection Assay Kit (S0175, Beyotime) according to the manufacture’s protocol.

### LDHA enzyme activity

Cells were lysed in lysis buffer and protein concentration was determined using BCA assay. 5 mg of total protein was added to the reaction solution containing 0.2 M Tris-HCl (pH 7.3), 0.05% bovine serum albumin, 2 mM pyruvate, 10 mM MgCl_2,_ and 20 mM NADH. The reduction in absorbance at 340 nm was measured due to the conversion of NADH to NAD^+^ using a Tecan spectrophotometer.

### Statistical analysis

Data are expressed as the means ± SD. All statistical analyses were performed using the GraphPad Prism5.0 software (La Jolla, CA). The significance of the differences between groups was estimated using Student’s *t* test, Mann–Whitney *U*-test, or one-way analysis of variance with Tukey post hoc analysis where appropriate. A probability level of 0.05 was chosen for statistical significance.

## Supplementary information

Supplemental material

## Data Availability

All data are available in the main text or Supplementary Materials.
