# Supplementary material for: Intracellular CYTL1, a novel tumor suppressor, stabilizes NDUFV1 to inhibit metabolic reprogramming in breast cancer
Source: Signal Transduct Target Ther. 2022 Feb 4;7:35. doi: 10.1038/s41392-021-00856-1 (PMC8813937; doi:10.1038/s41392-021-00856-1)
Supplement: Supplementary file 1 — Supplemental material [file 41392_2021_856_MOESM1_ESM.pdf]

Supplementary Materials for

Intracellular CYTL1, a novel tumor suppressor, stabilizes NDUFV1 to inhibit metabolic reprogramming in breast cancer

Wenwen Xue [1, a], Xin Li [1, a], Wuhao Li [1], Yixuan Wang [1], Chengfei Jiang [2], Lin Zhou [1], Jian Gao [1], Ying Yu [1], Yan Shen [1, *], Qiang Xu [a, *]

Correspondence to: shenyan@nju.edu.cn, molpharm@163.com

This PDF file includes:

Figures. S1 to S18

Supplementary Figure legends

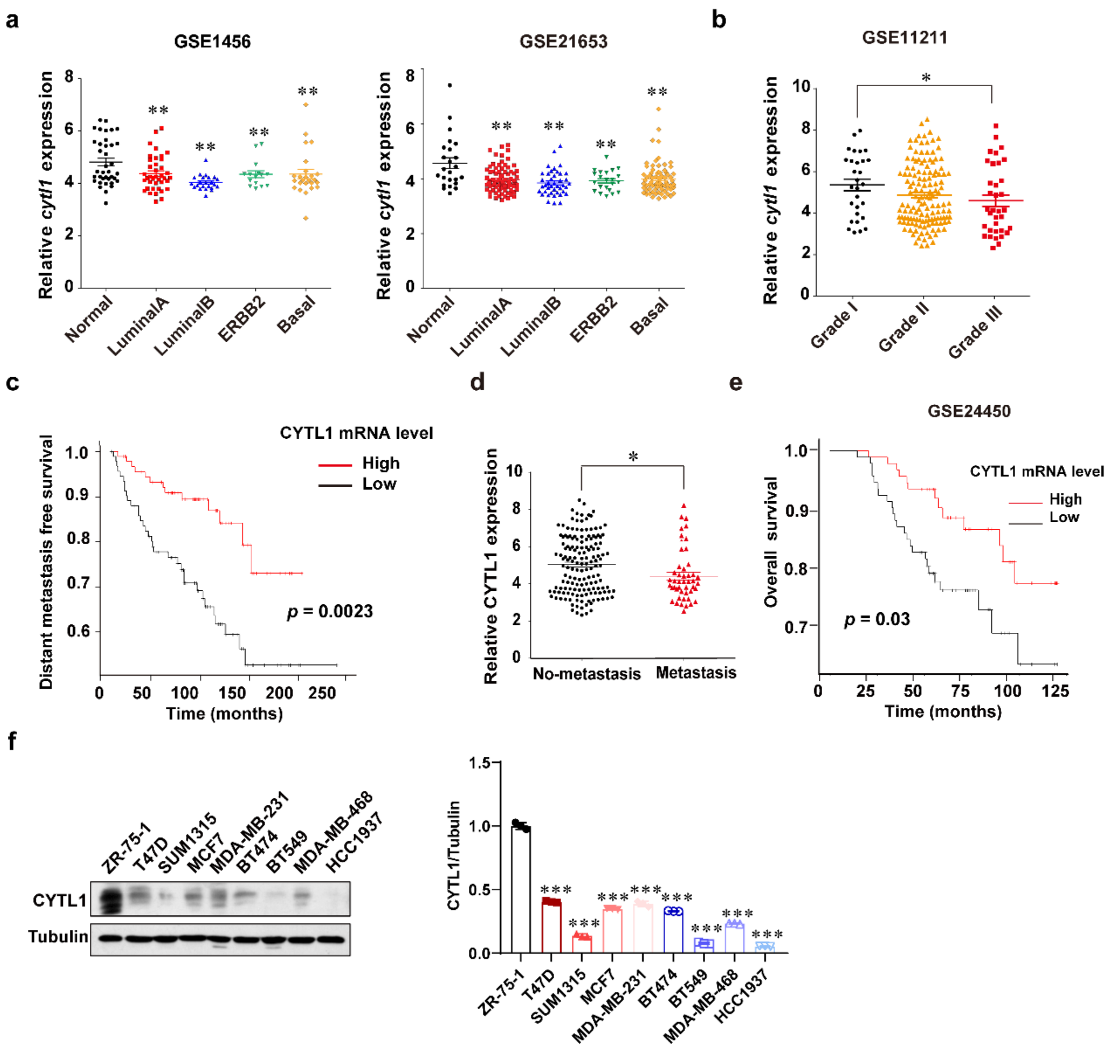

Figure S1 Low CYTL1 expression is strongly associated with human breast cancer progression. (a) Analysis of *cytl1* expression in different subtypes of human breast cancer based on dataset GSE1456 and GSE 21653 from GEO database. (b) Analysis of *cytl1* expression in different grades of human breast cancer based on dataset GSE11121. (c) Kaplan–Meier curve for DMFS based on CYTL1 mRNA levels. Breast cancer patients were divided into CYTL1 low expression group (n=100) and high expression group (n=100) according to the median value in dataset GSE11121. HR=2.715. (d) Analysis of the CYTL1 mRNA expression in metastasis and no-metastasis breast

cancer tissue based on dataset GSE11121. (e) Kaplan–Meier curve for overall survival based on CYTL1 mRNA levels in breast cancer patients. Patients were divided into a low CYTL1 expression group (n = 92) and a high expression group (n = 91) according to the median value in dataset GSE24450. HR=2.122. (f) The levels of CYTL1 protein expression were determined by western blot in a variety of breast cancer cell lines, including estrogen receptor positive breast cancer cell lines ZR-75-1, T47D, MCF7, BT474 and triple negative breast cancer cell lines SUM1315, MDA-MB-231, BT549, MDA-MB-468, HCC1937. Tubulin was used as a loading control. The densitometry of the immunoblots was performed with image J software and is presented in the histograms. The data are shown as the mean ± SD of three independent experiments. $^{*}P < 0.05$, $^{**} P < 0.01$, $^{***} P < 0.001$.

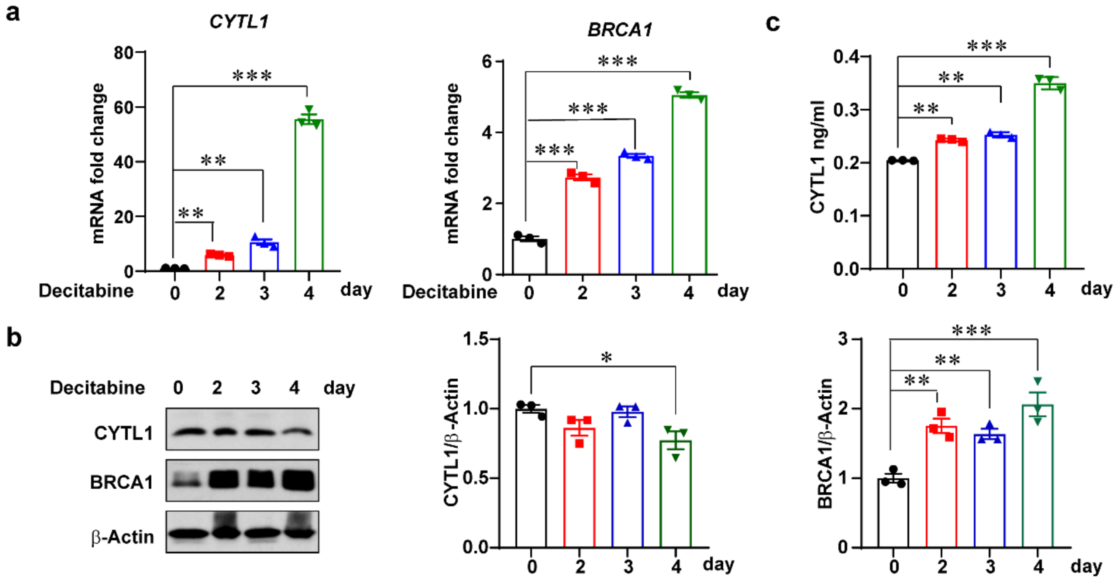

Figure S2 Breast cancer cells avidly lower the intracellular CYTL1 levels.

MDA-MB-231 cells were treated with 10 μM decitabine for the indicated time. (a) The mRNA levels of CYTL1 and BRCA1 were detected by real-time PCR. *β-Actin* was used as an internal control. (b) The protein levels of CYTL1 and BRCA1 were determined by western blot. β-Actin was used as a loading control. The densitometry of the immunoblots was performed with image J software and is presented in the histograms. The data are shown as the mean ± SD of three independent experiments. (c) The amount of CYTL1 in the culture supernatant were detected using ELISA assay. $^{*}P < 0.05$, $^{**}P < 0.01$, $^{***}P < 0.001$.

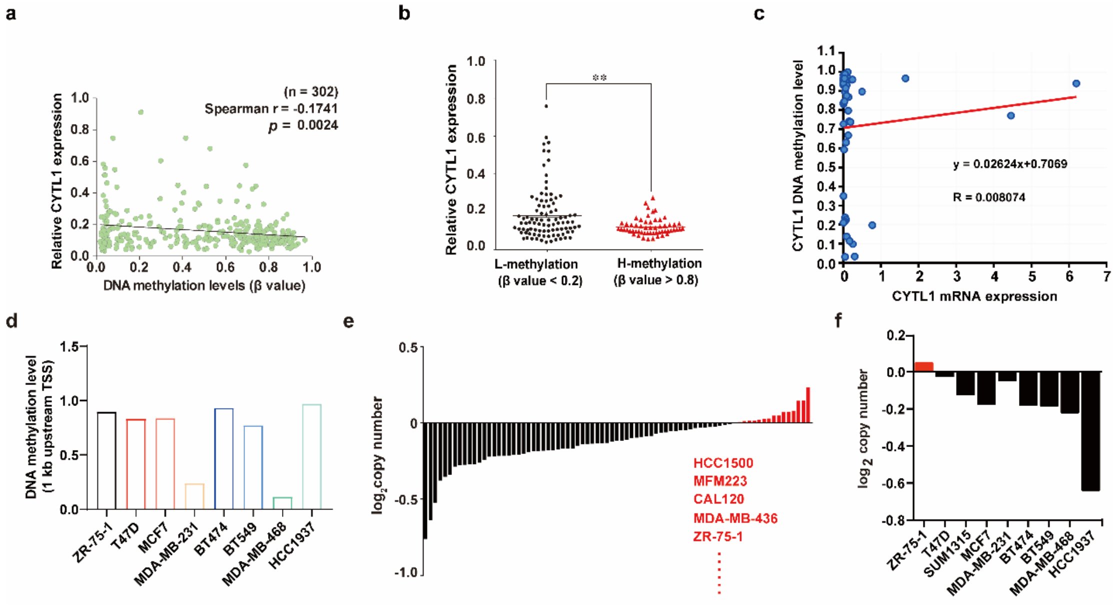

Figure S3 DNA copy number loss of CYTL1 may account for low CYTL1 expression in breast cancer cell lines. (a) Correlation analysis of *cytl1* expression and methylation levels in breast cancer tumors based on the TCGA database. (b) *cytl1* expression of tumors from breast cancer patients in low (L) or high (H) methylation groups classified according to β value from the TCGA database. (c) Correlation analysis of *cytl1* expression and methylation levels in breast cancer cell lines based on CCLE. (d) CYTL1 methylation levels of 8 breast cancer cell lines based on CCLE. (e) Copy number variation of breast cancer cell lines from CCLE. (f) Copy number of 9 breast cancer cell lines from CCLE. ** $P < 0.01$.

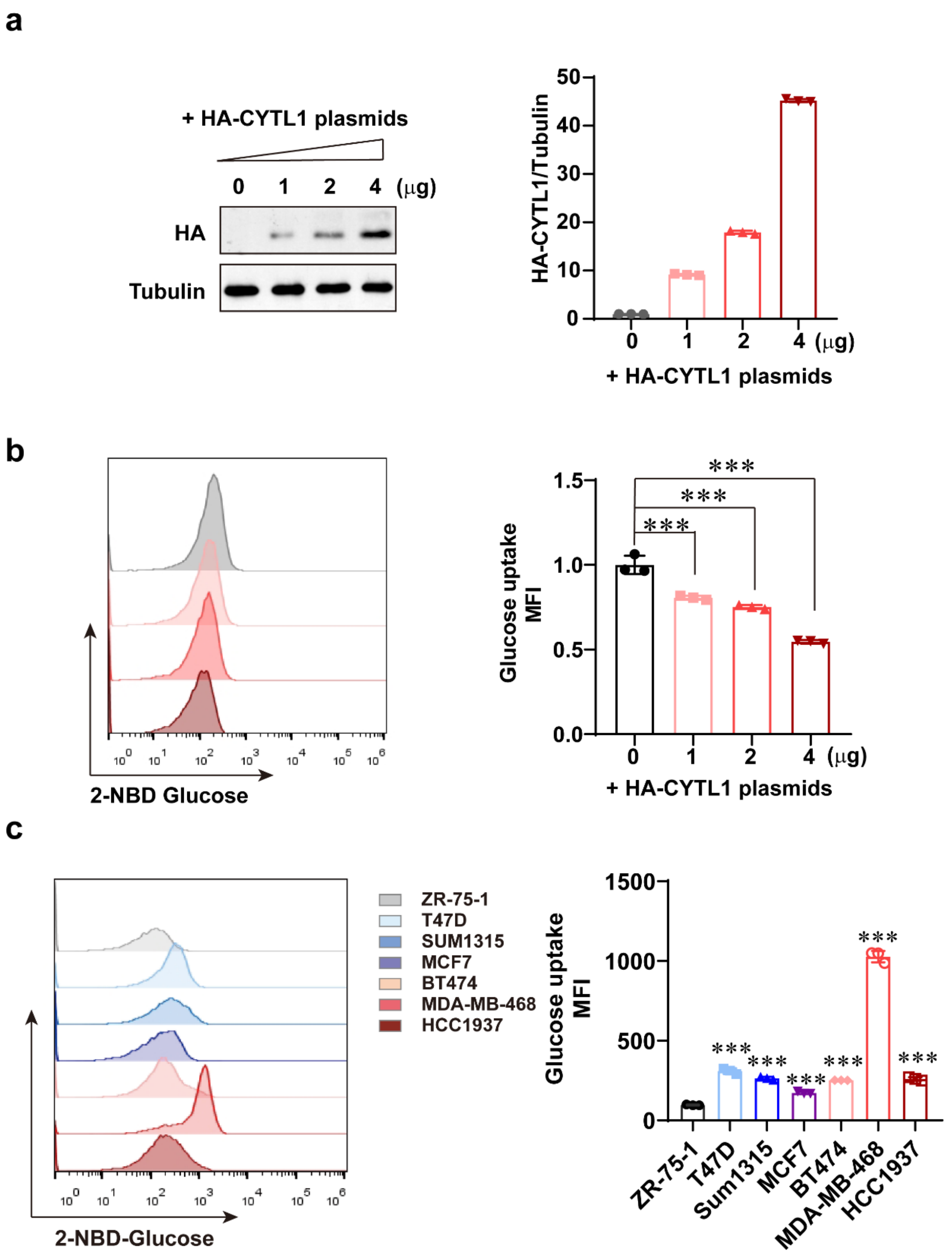

Figure S4 CYTL1 has an inhibitory effect on the glucose-uptake activity and the inhibitory intensity depends on the amount of CYTL1 in the cells. (a) MDA-MB-231 cells were transfected with various amounts of HA-tagged CYTL1 expressing plasmids (0-4 μg). 48h later, the CYTL1 protein expression was determined by western blot. Tubulin was used as a loading

control. The densitometry of the immunoblots was performed with image J software and is presented in the histograms. (b) Glucose-uptake activity in the resulting cells was detected by flow cytometry. The data are shown as the mean ± SD of three independent experiments. *** $P < 0.001$. (c) Glucose-uptake activity in a variety of breast cancer cell lines with different levels of CYTL1 expression. *** $P < 0.001$ compared with the glucose uptake in ZR-75-1 cells.

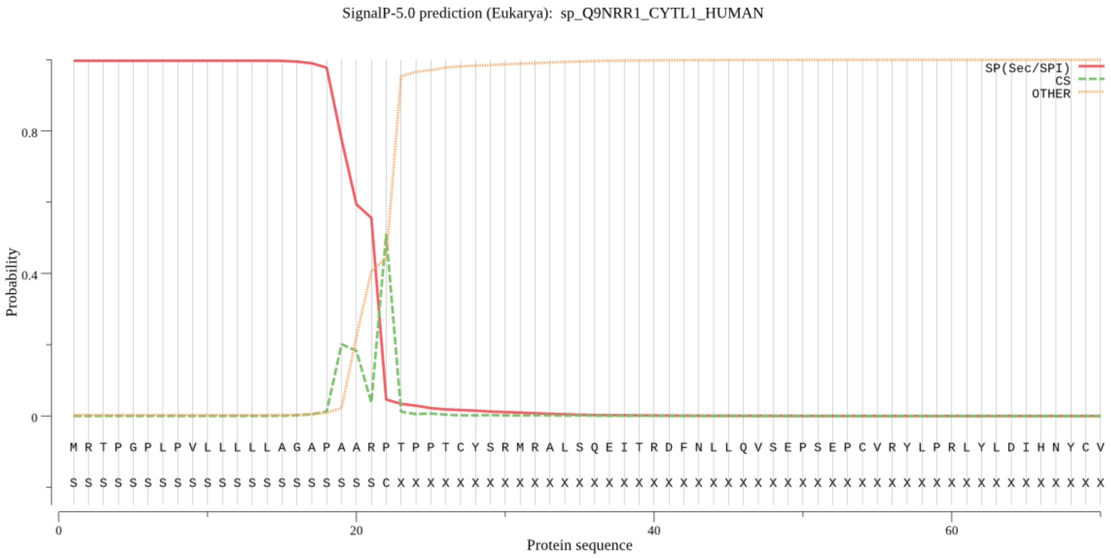

Figure S5 A signaling sequence from amino acid residue aa 1 to aa 22 in CYTL1. The signal peptide was predicted according to the web-link based bioinformatics analysis (http://www.cbs.dtu.dk/services/SignalP/).

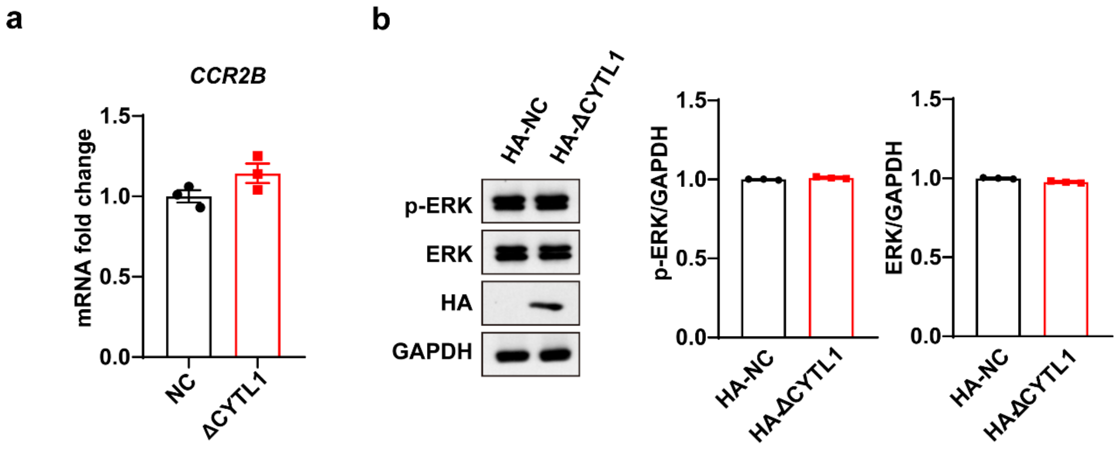

Figure S6 ΔCYTL1 has few effects on its potential receptor CCR2B and the downstream ERK signaling pathway. (a) The mRNA level of CCR2B was detected by real-time PCR in MDA-MB-231 cells transfected with HA-ΔCYTL1 expressing plasmids. GAPDH was used as an internal control. (b) The protein levels of p-ERK and total ERK were determined by western blot in MDA-MB-231 cells transfected with HA-ΔCYTL1 expressing plasmids. GAPDH was used as a loading control. The densitometry of the immunoblots was performed with image J software and is presented in the histograms. The data are shown as the mean ± SD of three independent experiments.

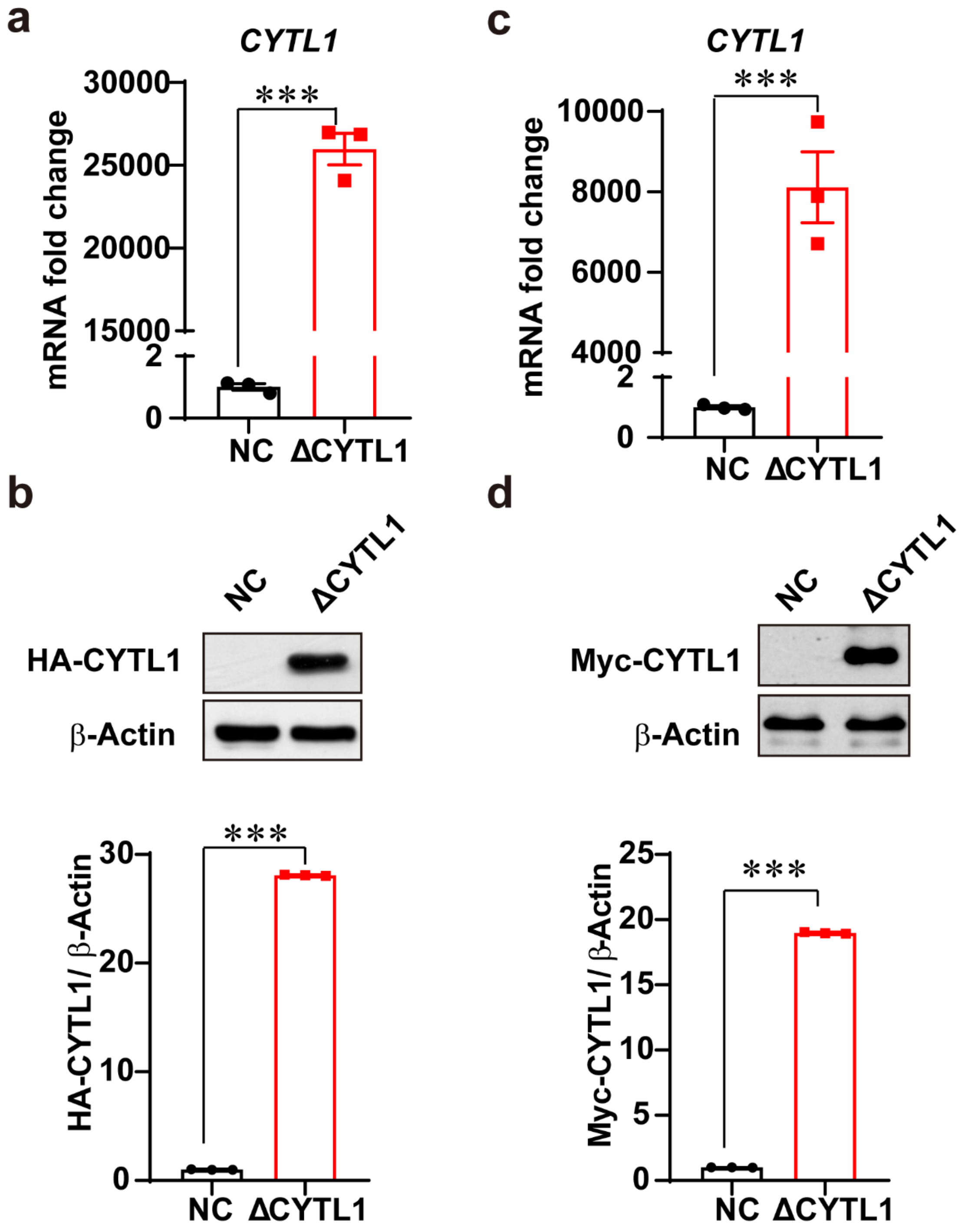

Figure S7 The efficiency of CYTL1 overexpression is confirmed in the stably transfected cells. Real-time PCR and western blot analysis confirming stable expression of ΔCYTL1 in (a, b) MDA-MB-231 cells transfected with lentiviral expression vectors and (c, d) E0771 cells transfected with the plasmids. β-Actin was used as a loading control. The

densitometry of the immunoblots was performed with image J software and is presented in the histograms. The data are shown as the mean ± SD of three independent experiments. *** $P < 0.001$.

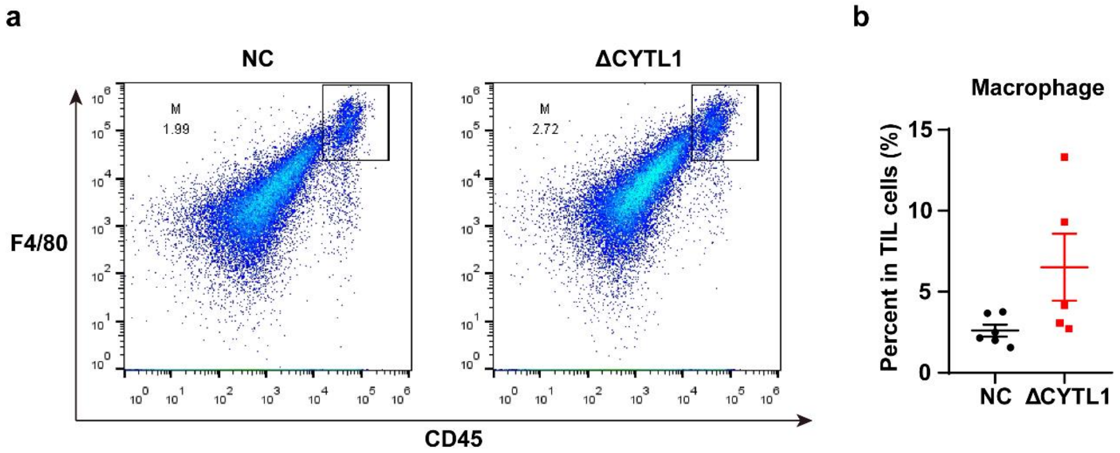

Figure S8 ΔCYTL1 has no chemotactic effect on macrophages in mice.

Female C57BL/6 mice were subjected to orthotopic injection with E0771 cells stably expressing ΔCYTL1. The tumor-infiltrating leukocytes (TIL) were isolated from tumor tissues and analyzed by flow cytometry. (a) Representative analysis images of the F4/80^{+} CD45^{+} macrophages. (b) Quantitative analysis of the percentage of macrophages among TIL.

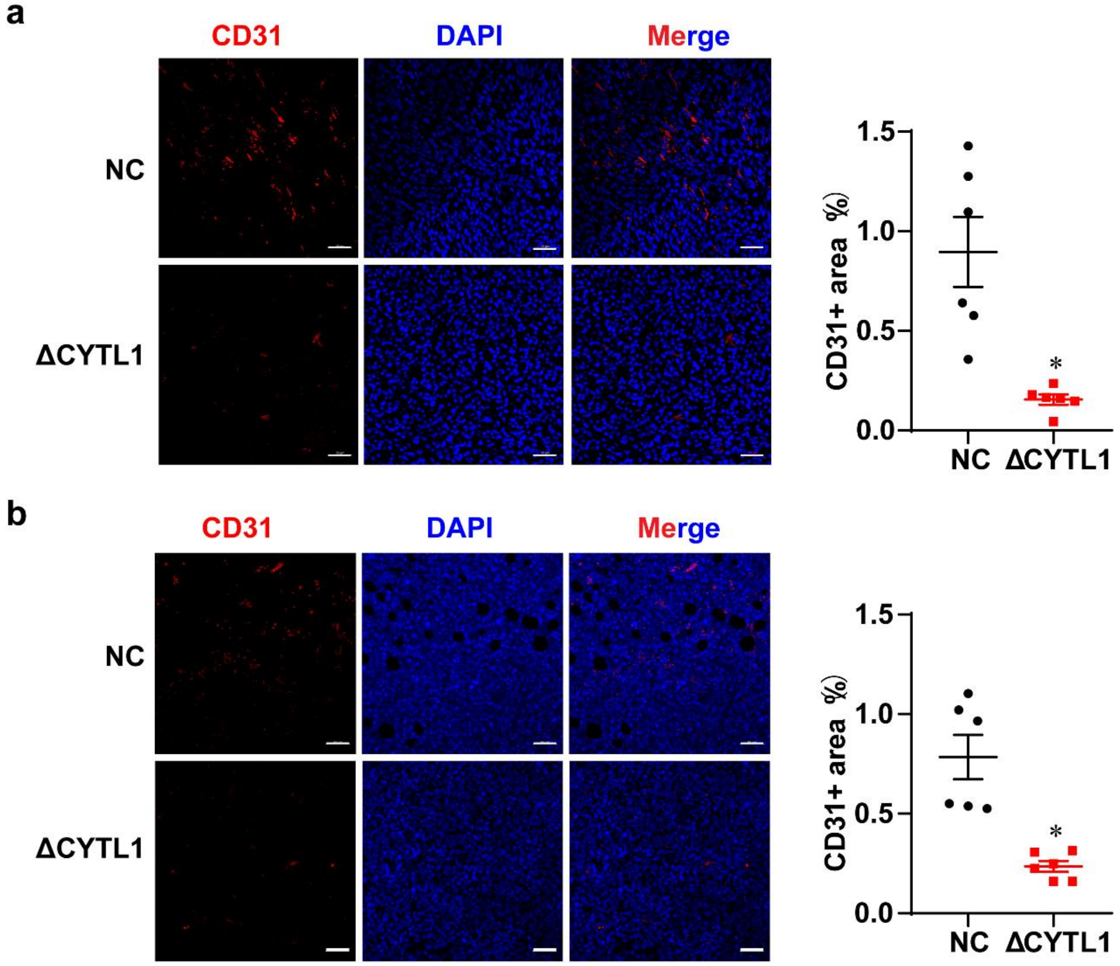

Figure S9 ΔCYTL1 reduced angiogenesis in breast cancer models. Female nude mice and C57BL/6 mice were subjected to orthotopic injection with (a) MDA-MB-231 cells and (b) E0771 cells stably expressing ΔCYTL1, respectively. Tumor tissue sections were stained with an antibody specific for CD31. Scale bar: 50 μm. $^{*}P < 0.05$.

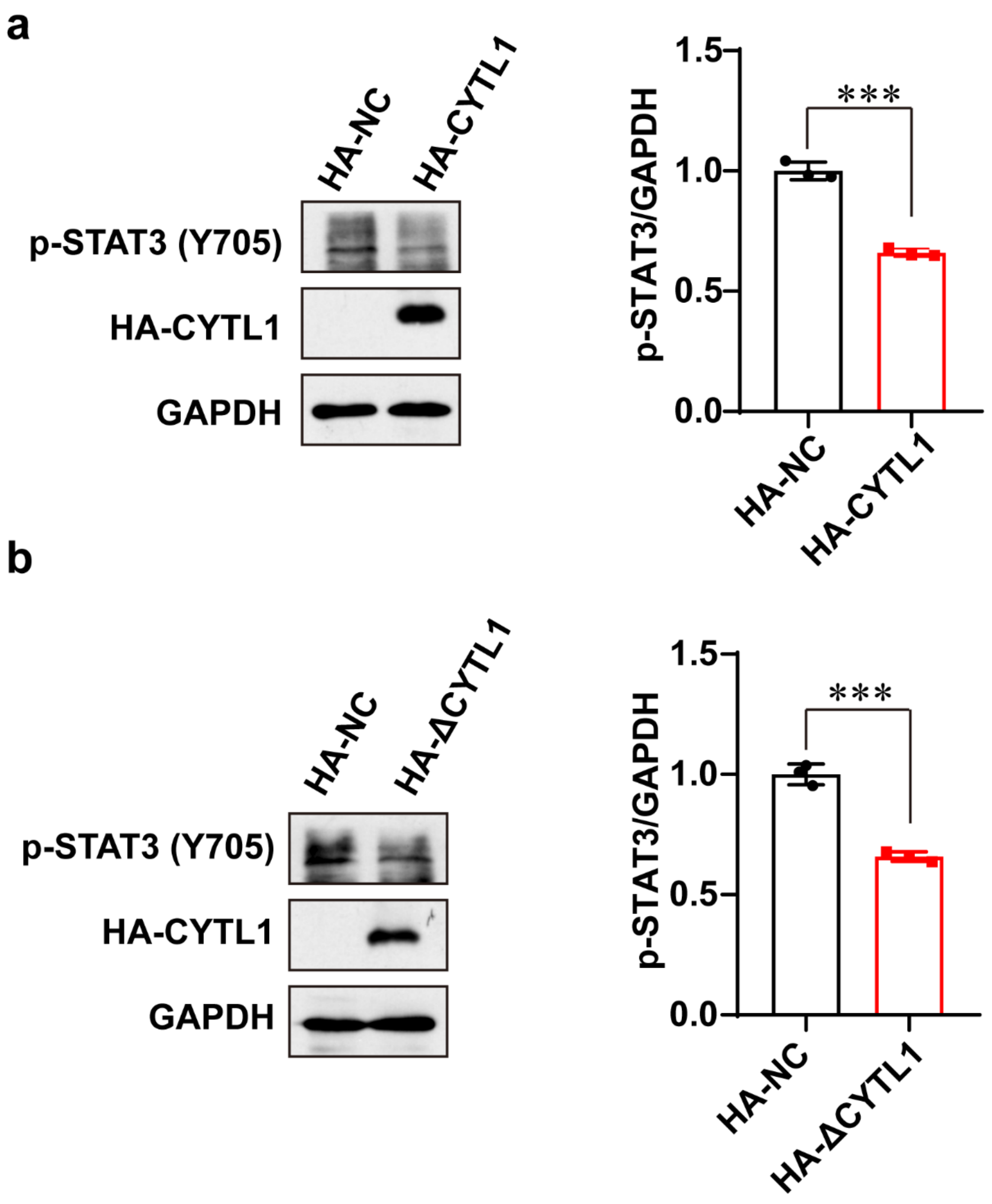

Figure S10 Ectopic expression of both CYTL1 and ΔCYTL1 decreased STAT3 phosphorylation. The protein level of p-STAT3 was determined by western blot in MDA-MB-231 cells transfected with HA-CYTL1 (a) or HA-ΔCYTL1 (b) expressing plasmids. GAPDH was used as a loading control. The densitometry of the immunoblots was performed with image J software and is presented in the histograms. The data are shown as the mean ± SD of three independent experiments. *** $P < 0.001$.

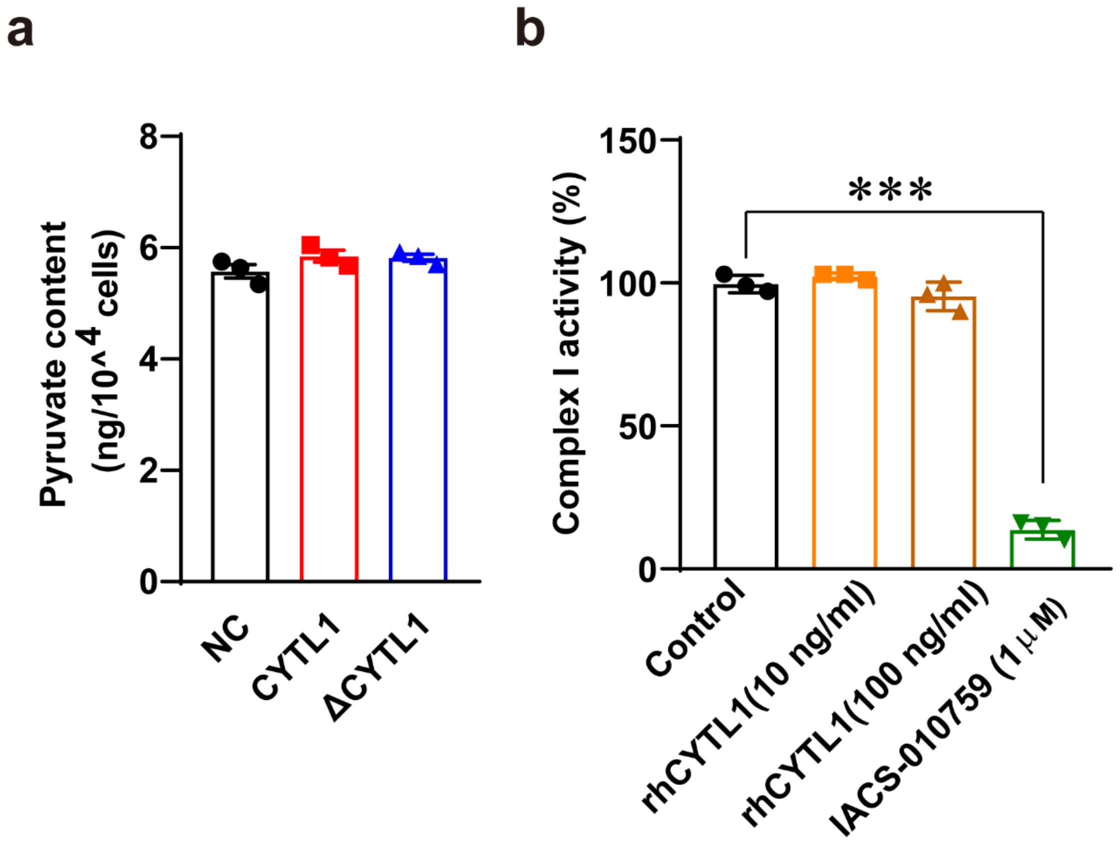

Figure S11 CYTL1 has no effect on pyruvate production and complex I activity in breast cancer cells. (a) Pyruvate level in cell lysates from MDA-MB-231 cells transfected with HA-tagged CYTL1 or ΔCYTL1 expressing plasmids was determined using pyruvate assay kit. (b) Bivine heart mitochondria was treated in the presence of the indicated concentrations of rhCYTL1 or IACS-010759 and then complex I activity was determined using complex I activity assay kit. *** $P < 0.001$.

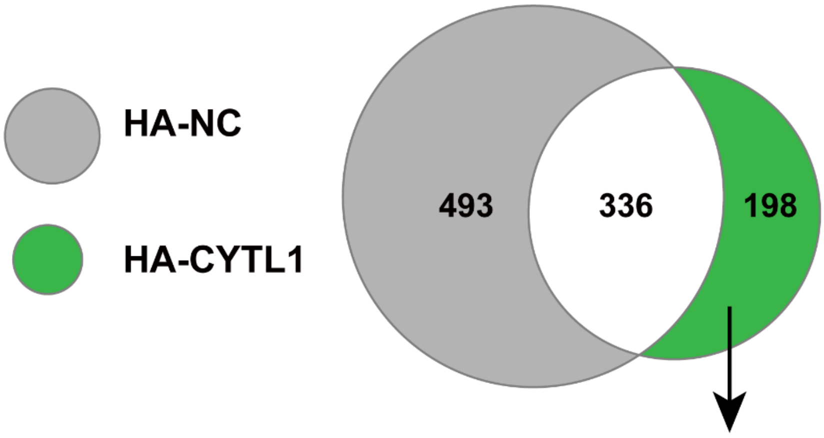

Name	Peptides(95%)
KRT1	42
FLNA	29
HEL-S-124m	26
ANXA2	13
FLNC	13
PGAM1	13
ACTN1	11
P4HB	10
FLNB	9
PRDX1	9
PTBP1	8
PSMA2	7
TLN1	7
YWHAZ	7
YWHAG	7
HEL-S-5a	6
RPL10A	6
HNRNPK	5
HEL-S-47e	5
VDAC2	4
VDAC3	4
PSMA1	4
MYOF	4
GOT2	4
CAD	4
KRT6C	4
EIF3L	3
RANBP5	3
MTAP	3
ARPC4	3

Name	Peptides(95%)
H2AFJ	3
MYL6	3
DYNC1H1	3
DLST	3
PSMA7	3
PSMA6	3
EIF3D	3
DDOST	3
DKFZp686G04235	3
SFPQ	3
STOML2	3
PSMB3	3
NDUFV1	3
TWF1	3
PUF60	3
NCL	3
GPD2	2
PSMD6	2
UBE2I	2
EIF2S3	2
RPS4X	2
FKBP1A	2
COPS7A	2
NUP155	2
RPS13	2
RAB10	2
NONO	2
PPID	2
ANXA3	2
EIF3B	2

Figure S12 Pull-down experiments coupled with mass spectrometry identification reveals NDUFV1 as a potential CYTL1-interacting protein. Proteins were immunopurified from MDA-MB-231 cells transfected with HA-NC or HA-CYTL1 expressing plasmids using anti-HA antibody, and then were applied to mass spectrometry. 60 candidate proteins with high ranking are listed.

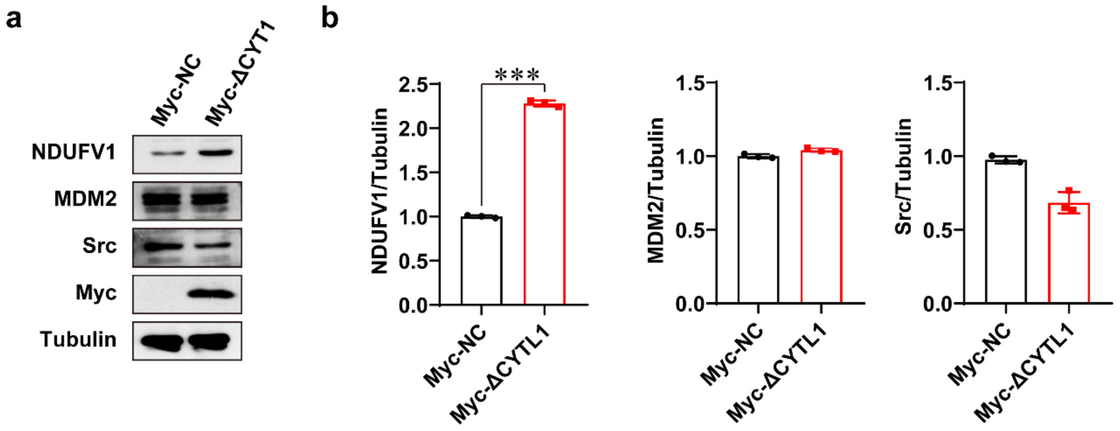

Figure S13 The level of NDUFV1 was remarkably increased in E0771 cells stably expressing ΔCYTL1. The indicated proteins were detected by western blot in E0771 cells stably expressing ΔCYTL1. Tubulin was used as a loading control. The densitometry of the immunoblots was performed with image J software and is presented in the histograms. The data are shown as the mean ± SD of three independent experiments. *** $P < 0.001$.

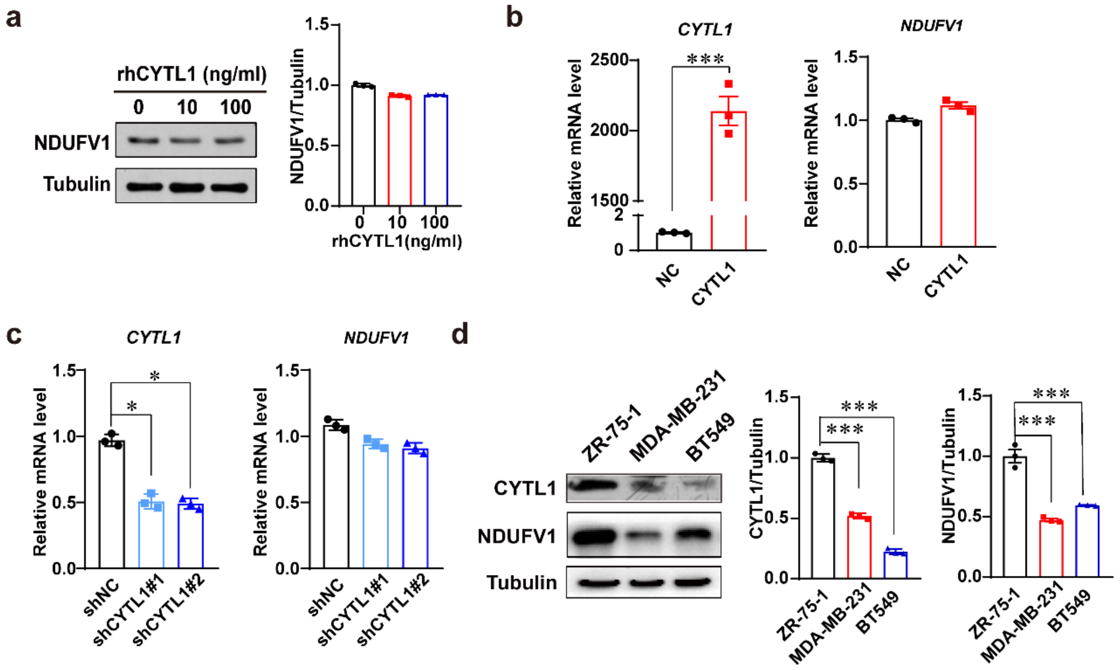

Figure S14 Intracellular CYTL1 regulates NDUFV1 protein expression at the posttranslational level. (a) The protein level of NDUFV1 was determined by western blot in MDA-MB-231 cells after treatment with rhCYTL1 at the indicated concentrations for 72 h. (b, c) The mRNA levels of CYTL1 and NDUFV1 were detected by real-time PCR in MDA-MB-231 cells transfected with (b) HA-CYTL1 expressing plasmids or (c) the indicated shRNAs. (d) The protein levels of CYTL1 and NDUFV1 in the indicated breast cancer cell lines. Tubulin was used as a loading control. The densitometry of the immunoblots was performed with image J software and is presented in the histograms. The data are shown as the mean ± SD of three independent experiments. *$P < 0.05$, *** $P < 0.001$.

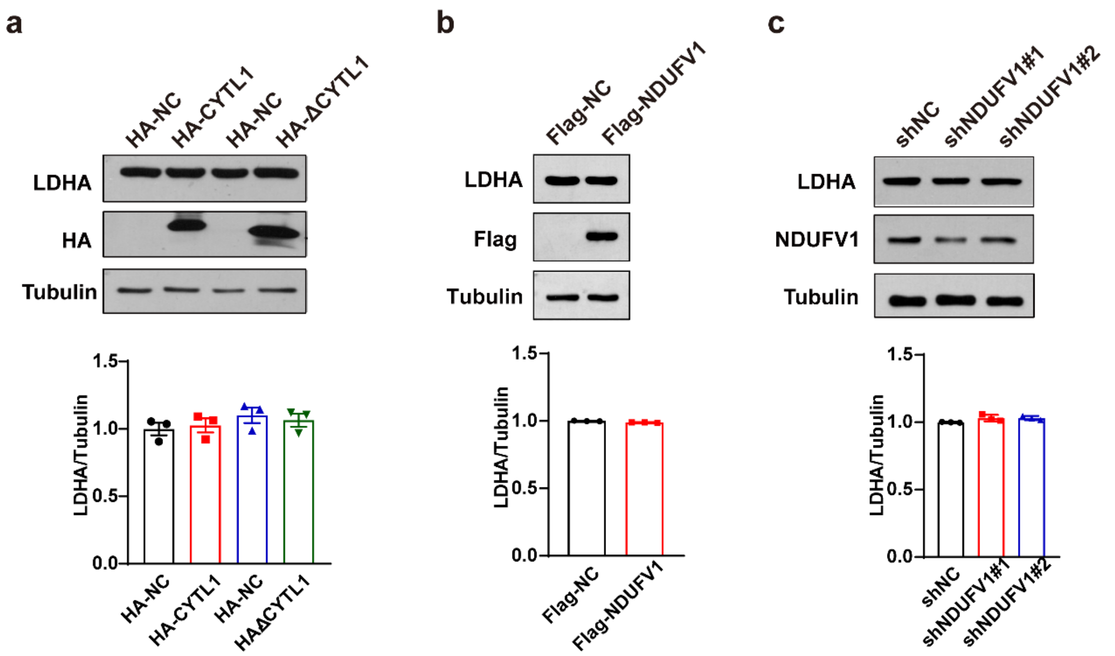

Figure S15 Neither CYTL1 nor NDUFV1 abundance has any effect on LDHA protein expression. LDHA protein expression was determined by western blot in MDA-MB-231 cells transfected with (a) HA-CYTL1 or HA-ΔCYTL1 expressing plasmids, (b) Flag-NDUFV1 expressing plasmids or (c) the shRNAs against NDUFV1. Tubulin was used as a loading control. The densitometry of the immunoblots was performed with image J software and is presented in the histograms. The data are shown as the mean ± SD of three independent experiments.

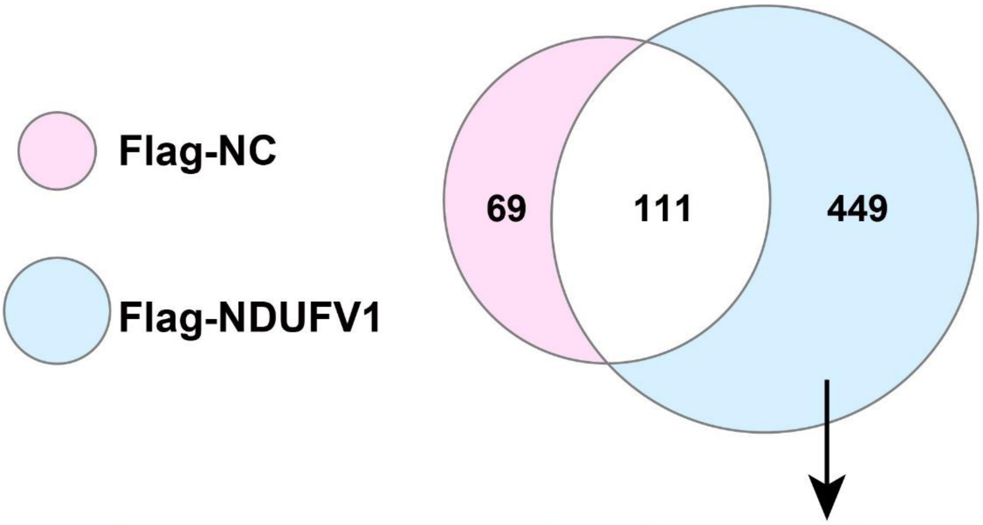

Name	Peptides(95%)
Beta actin	35
TUBB2C	33
NDUFV1	24
Tubulin	18
NDUFV2	18
TUBA1A	18
PRDX1	12
DECR1	11
XRCC6	10
HMGCL	10
UEVLD	9
RARS	9
RPL7A	8
AR	8
RPS4X	8
ACTN4	8
PABPC1	8
MYO1C	7
IRF2BP2	7
EEF2	7

Name	Peptides(95%)
ACTN1	7
EL52	7
EEF1A2	7
ARHGAP10	6
HSD17B10	6
CAPZA1	6
C2orf33	6
GNAS	6
MYL12A	6
YBX1	6
DBT	6
SLC3A2	5
IVNS1ABP	5
TPD52L2	5
CCDC6	5
ATP50	5
DLST	5
**SRC**	4
HEL-215	4
RPS2	4

Figure S16 Pull-down experiments coupled with mass spectrometry identification reveals Src as a potential NDUFV1-interacting protein. Proteins were immunopurified from BT549 cells transfected with Flag-NC or Flag-NDUFV1 expressing plasmids using anti-Flag antibody, and then were applied to mass spectrometry. 40 candidate proteins with high ranking are listed.

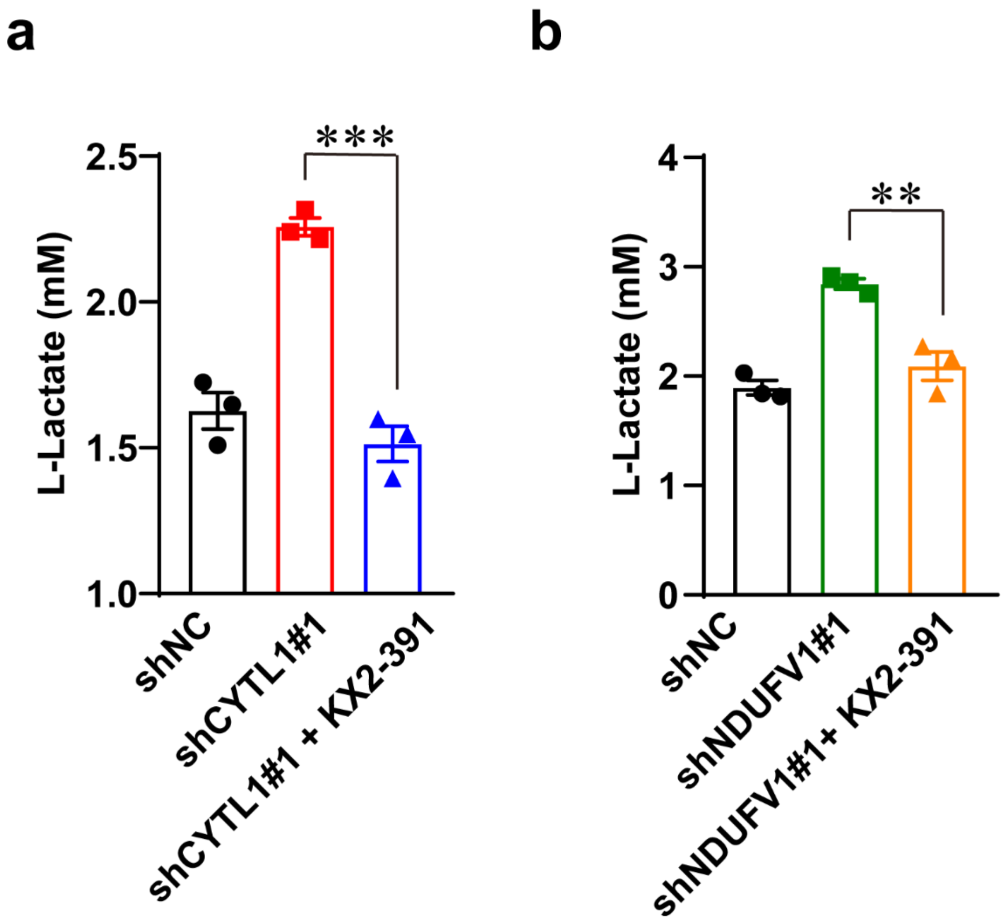

Figure S17 Pharmacological Src inhibition suppressed the increase of lactate production in MDA-MB-231 cells with CYTL1 or NDUFV1 silence. Lactate production levels in MDA-MB-231 cells transfected with (a) CYTL1 shRNA#1 or (b) NDUFV1 shRNA#1 were detected in the absence or presence of KX2-391 by a spectrophotometer and normalized to the cell number. The data are shown as the mean ± SD of three independent experiments. ** *P* < 0.01, *** *P* < 0.001.

Figure 1

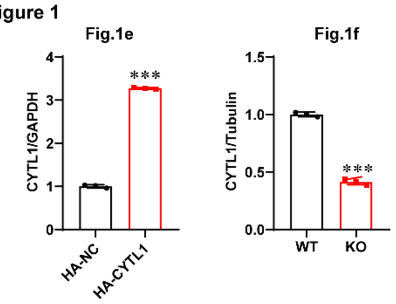

Figure 4

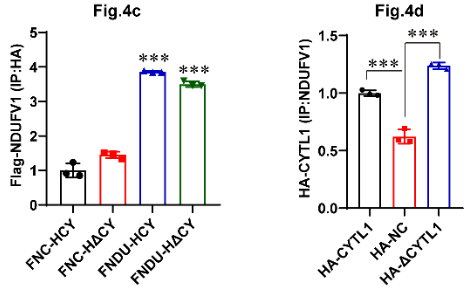

Figure 5

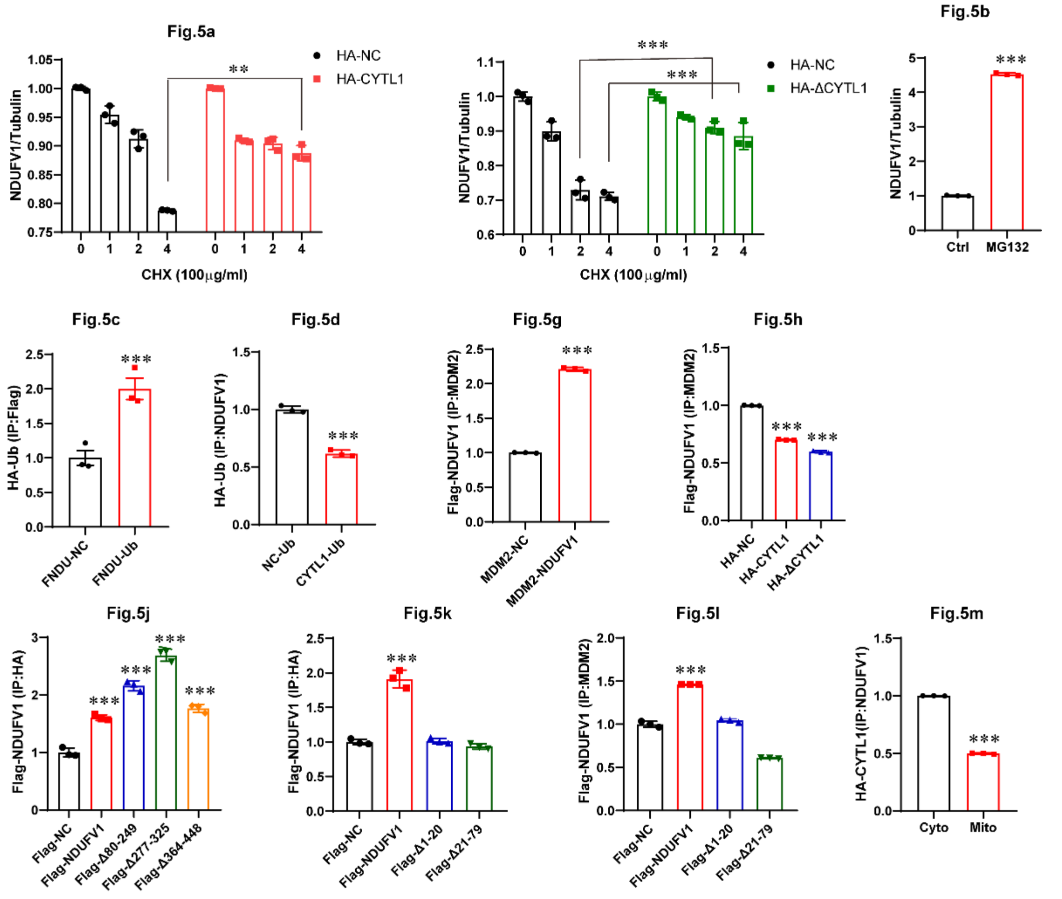

Figure 6

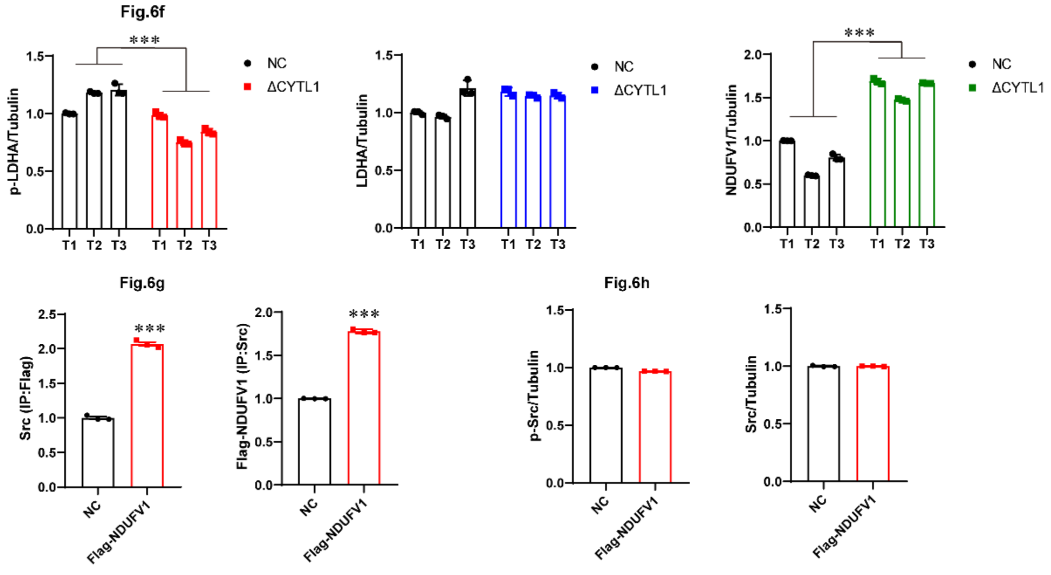

Figure S18 The Immunoblots are required to quantify and represent graphically. The densitometry of the immunoblots was performed with image J software and is presented in the histograms. The data are shown as the mean ± SD of three independent experiments. ** $P < 0.01$, *** $P < 0.001$.
